# ZSCAN5B and primate-specific paralogs bind RNA polymerase III genes and extra-TFIIIC (ETC) sites to modulate mitotic progression

**DOI:** 10.18632/oncotarget.12508

**Published:** 2016-10-06

**Authors:** Younguk Sun, Huimin Zhang, Majid Kazemian, Joseph M. Troy, Christopher Seward, Xiaochen Lu, Lisa Stubbs

**Affiliations:** ^1^ Institute for Genomic Biology, University of Illinois at Urbana-Champaign, Urbana, IL, USA; ^2^ Department of Cell and Developmental Biology, University of Illinois at Urbana-Champaign, Urbana, IL, USA; ^3^ Illinois Informatics Program, University of Illinois at Urbana-Champaign, Urbana, IL, USA; ^4^ Laboratory of Molecular Immunology and the Immunology Center, National Heart, Lung, and Blood Institute, National Institutes of Health, Bethesda, MD, USA

**Keywords:** zinc finger transcription factor, primate-specific duplication, RNA Polymerase III transcription, chromatin architecture, cell cycle, Chromosome Section

## Abstract

Mammalian genomes contain hundreds of genes transcribed by RNA Polymerase III (Pol III), encoding noncoding RNAs and especially the tRNAs specialized to carry specific amino acids to the ribosome for protein synthesis. In addition to this well-known function, tRNAs and their genes (tDNAs) serve a variety of other critical cellular functions. For example, tRNAs and other Pol III transcripts can be cleaved to yield small RNAs with potent regulatory activities. Furthermore, from yeast to mammals, active tDNAs and related “extra-TFIIIC” (ETC) loci provide the DNA scaffolds for the most ancient known mechanism of three-dimensional chromatin architecture. Here we identify the ZSCAN5 TF family - including mammalian ZSCAN5B and its primate-specific paralogs - as proteins that occupy mammalian Pol III promoters and ETC sites. We show that ZSCAN5B binds with high specificity to a conserved subset of Pol III genes in human and mouse. Furthermore, primate-specific ZSCAN5A and ZSCAN5D also bind Pol III genes, although ZSCAN5D preferentially localizes to MIR SINE- and LINE2-associated ETC sites. ZSCAN5 genes are expressed in proliferating cell populations and are cell-cycle regulated, and siRNA knockdown experiments suggested a cooperative role in regulation of mitotic progression. Consistent with this prediction, *ZSCAN5A* knockdown led to increasing numbers of cells in mitosis and the appearance of cells. Together, these data implicate the role of ZSCAN5 genes in regulation of Pol III genes and nearby Pol II loci, ultimately influencing cell cycle progression and differentiation in a variety of tissues.

## INTRODUCTION

Most eukaryotic genes are transcribed by RNA Polymerase II (RNA Pol II) and its attendant transcriptional machinery. However, a significant number of non-coding RNAs (ncRNAs), including tRNAs, 5SRNA, U6 small nuclear RNA, and other essential transcripts, depend on the activity of RNA Polymerase III (Pol III) (reviewed in [1]). Pol III promoters exist in three major types, depending on their locations relative to the transcription start site (upstream of or internal to the transcript) and distinct sets of general transcription factors (TFs) - TFIIIA, TFIIIB and TFIIIC. Despite their different structures, Pol II and Pol III promoters share many features. For example, active promoters of both gene types are marked by similar configurations of modified histones [2]; furthermore, several TFs classically identified as Pol II regulators, such as MYC, P53 and MAF1, also regulate the expression of Pol III genes (reviewed in [3]).

Because they are central to basic cellular functions including translation, Pol III transcripts are essential to cellular survival. However, tRNAs and other Pol III transcripts also play a variety of other critical, noncanonical roles. Notably, active tDNAs are key participants in the most ancient known mechanism of chromatin organization, clustering together within the nucleus to serve chromatin barrier, insulator and other regulatory functions [4-7]. Most vertebrate tDNAs are organized in genomic clusters at syntenically homologous positions, thus providing a stable and conserved framework for chromatin structure [8]. However, not all TFIIIC binding sites are so highly conserved. In particular, extra-TFIIIC, or ETC sites, also called “chromatin organizing clamps” in yeast, interact with each other and with tDNA to influence chromatin architecture [9, 10]. In mammals, Pol III and TFIIIC binding sites include transposable elements such as MIRs, ALUs and other SINE subfamilies that were originally derived from Pol III transcription units [11]. These lineage-specific transposable elements (TEs) greatly outnumber the conserved tDNA sites in mammalian genomes.

Furthermore, tRNAs and other Pol III transcripts, including the RNA component of RNase MRP (encoded by *RMRP)*, are processed to generate small RNAs with diverse roles in cell proliferation and differentiation [12, 13]. The expression of individual Pol III genes and thus, their participation in both traditional and extra-translational functions varies according to cell type, cellular state and developmental stage [14-18], and in different cell types [2, 16, 18-20]. The selection of specific tDNAs for expression or silencing has direct developmental consequences [21], and indirect effects on the transcription of nearby Pol II genes [6, 19, 22, 23]. Possibly relevant to this indirect effect, tRNA expression plays a decisive role in nuclear clustering and thus likely, the selection of alternative anchors for chromatin loops [8, 10, 23, 24].

Here we report the DNA-binding functions of ZSCAN5B, a protein encoded by a unique eutherian SCAN domain-containing zinc finger (SCAN-ZNF) gene, and two human paralogs (*ZSCAN5A* and *ZSCAN5D*) that arose in early primate lineages. Primate *ZSCAN5A* and *ZSCAN5D* have diverged from *ZSCAN5B* in both sequence and tissue-specific expression patterns, but share expression in dividing cell populations with a distinct peak around the time of mitosis. Combining chromatin immunoprecipitation sequencing (ChIP-seq) with analysis of gene expression after siRNA knockdown, we discovered that Pol III genes and ETCs are the strongly preferred binding sites for mouse Zscan5b and human ZSCAN5 proteins, and that ZSCAN5 gene knockdown alters expression of the Pol III genes. We also documented the dysregulation of nearby polymerase II (Pol II)-transcribed genes that predict cooperative functions in control of mitosis and cell fate decisions in multiple tissues. Consistent with these predictions, stable knockdown of *ZSCAN5A* led to the accumulation of cells in mitosis and aneuploidy in cultured human cells. Based on these data, we conjecture that *ZSCAN5B* evolved in eutherians to directly modulate the activities of ancient Pol III gene activities including secondary effects on nearby Pol II genes. Further we hypothesize that in primates, *ZSCAN5A* and *ZSCAN5D* evolved to independently extend these regulatory activities to a wider range of TFIIIC binding sites, including those carried by MIR and L2 repeat-associated ETCs.

## RESULTS

### The ZSCAN5 family arose by duplication of conserved *Zscan5b* in early primate history

**Figure 1:**
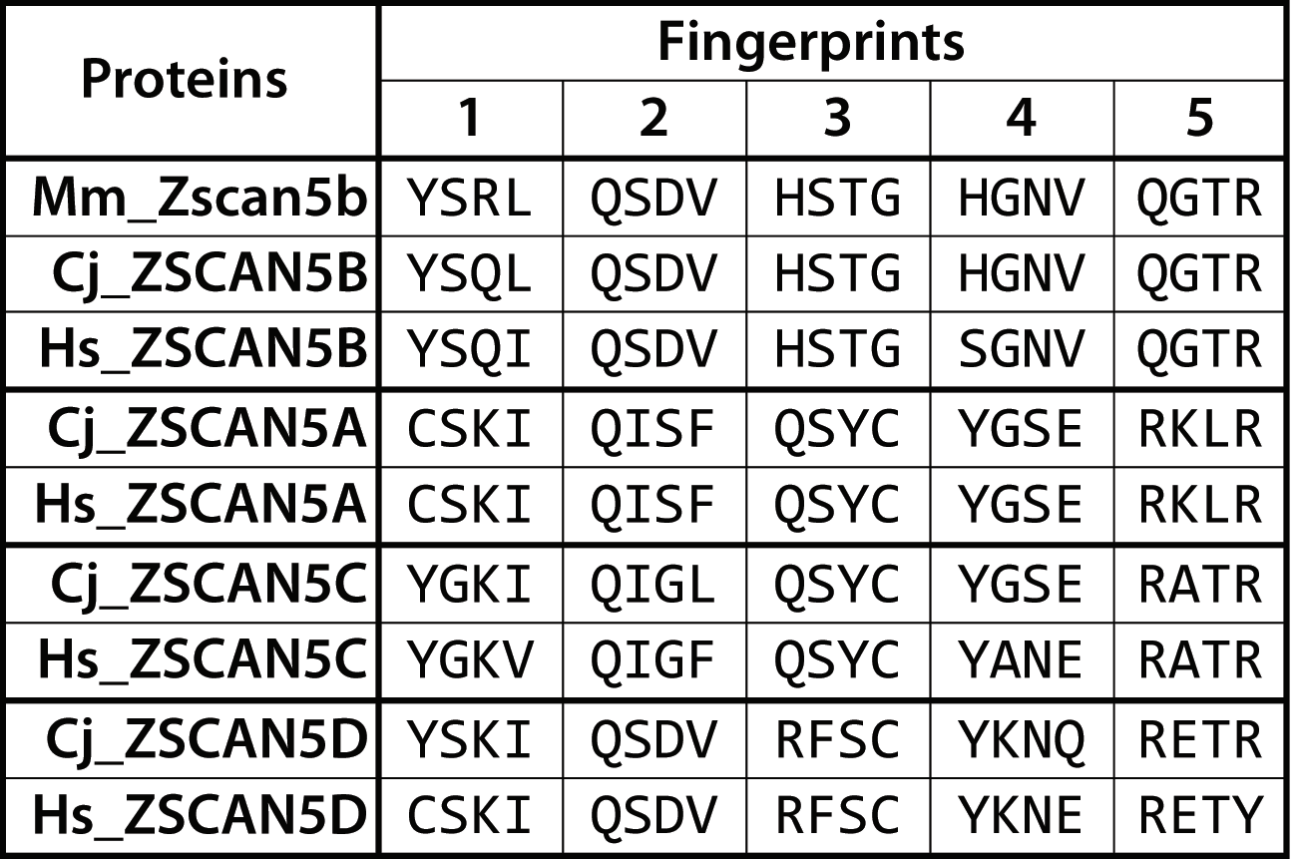
Diverged DNA-binding “fingerprint” patterns for primate-specific ZSCAN5 proteins Amino acid residues corresponding to DNA-binding positions (1-, 2, 3, and 6 relative to the alpha helix) in each of the five zinc fingers in ZSCAN5 proteins predicted from mouse (Mm), human (Hs), and marmoset (Cj) genomes are shown aligned in the C- > N terminal order of the zinc fingers in each gene. Mouse and other mammals contain a single gene, *Zscan5b*, which was duplicated in early primate history to generate three new gene copies: *ZSCAN5A, ZSCAN5C*, and *ZSCAN5D* (Mm_Zscan5b: ENSMUSG00000058028, Cj_ZSCAN5B: ENSCJAG00000020279, Hs_ZSCAN5B: ENSG00000197213, Cj_ZSCAN5A: ENSCJAG00000037918, Hs_ZSCAN5A: ENSG00000131848, Cj_ZSCAN5C: ENSCJAG00000020275, Hs_ZSCAN5C: ENSG00000204532, Cj_ZSCAN5D: ENSCJAG00000014787, Hs_ZSCAN5D: ENSG00000267908). The fingerprints of the novel duplicates have diverged relative to the ancestral gene copy, but once generated, these patterns have been conserved in primate species.

*Zscan5b* is a unique gene in mouse and most other eutherian genomes, but primate genomes contain four very closely related gene copies, annotated as human *ZSCAN5A, ZSCAN5B, ZSCAN5C* and *ZSCAN5D* [25]. Human *ZSCAN5B* is the ortholog of the single mouse gene as confirmed by overall sequence similarity as well as the alignment of the DNA-binding amino acids of each zinc finger (corresponding to amino acids −1, 2, 3, and 6 relative to the alpha-helix) [26-28] (Figure 1). For simplicity, as we have in a recent paper [29], we will refer to this pattern of DNA-binding amino acid quadruplets as a protein's “fingerprint” in the following discussion.

Mouse, marmoset and human ZSCAN5B proteins share almost identical fingerprints, but the three primate-specific paralogs have diverged from the parental gene in fingerprint patterns. After an initial phase of divergence in early primate history, these patterns have been very well conserved (Figure 1). In contrast to the zinc fingers, the SCAN domains of the four human ZSCAN5 family members are nearly identical (95-98% identity between members; not shown). Since SCAN mediates protein dimer formation [30, 31], this suggests that ZSCAN5 family members could form homo/heterodimers, cooperating in different combinatorial patterns in tissues and cell types where they are co-expressed.

We found orthologs of all four human genes in the marmoset genome but identified only a unique *ZSCAN5B*-related copy in other eutherian genomes and in genomes of more primitive primates such as *Galago* (not shown). Available evidence therefore indicates that *ZSCAN5A, ZSCAN5C*, and *ZSCAN5D* arose in the ancestors of new world monkeys and have been conserved in higher primates after a rapid period of divergence.

### ZSCAN5 paralogs display overlapping but unique patterns of expression

#### Overlapping but distinct patterns of tissue-specific expression in human tissues

**Figure 2:**
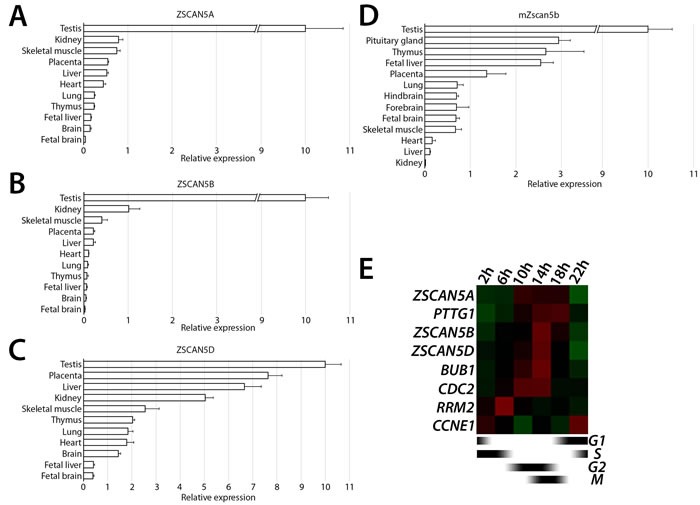
Tissue- and cell cycle stage-specific expression ZSCAN5 family genes Relative transcript levels measured in cDNA prepared from total tissue RNA for **A.** human *ZSCAN5A*, **B.** *ZSCAN5B*, **C.** *ZSCAN5D*, and paralogous gene for **D.** mouse *Zscan5b* by qRT-PCR. Relative expression in other tissues was normalized to expression level in testis, which was set as 10. Error bars correspond to the variance between experimental triplicates. **E.** Heat map showing the patterns of expression for ZSCAN5A, ZSCAN5B, and ZSCAN5D in HEK-293 cells collected at different times after release from a double thymidine block to synchronize cells at early S-phase. Expression levels of *CCNE1*, *RRM2*, *CDC2*, *BUB1* and *PTTG1* were measured on the same cDNA samples to monitor progression through the cell cycle.

Publicly available data indicated that both human and mouse ZSCAN5 genes are expressed at high levels in testis but at very low levels in most other adult tissues. To further examine expression profiles of ZSCAN5 family members, we used quantitative reverse transcript PCR (qRT-PCR) to measure transcript levels of the unique mouse *Zscan5b* and all four human ZSCAN5 genes in panels of RNA derived from adult and embryonic tissues (Figure 2A-2D). *ZSCAN5C* transcripts were not detected, or were detected near background levels, in every tissue we tested (not shown). As expected, mouse *Zscan5b* and all three expressed human paralogs were detected at highest levels in adult testis; in adult mouse, we also found *Zscan5b* transcript in thymus, fetal liver and placenta with lower levels of expression in brain, lung and skeletal muscle. Quantitative RT-PCR across a similar panel of tissues revealed significant overlaps, but also showed that each of the human duplicates displays distinct features of gene expression compared to each other and to the unique mouse gene; *ZSCAN5A* and *ZSCAN5B* expression patterns overlap significantly, while the *ZSCAN5D* expression pattern has diverged most significantly from the other human paralogs and the mouse gene.

#### Human ZSCAN5 genes display a peak of mitotic expression

*ZSCAN5A* was identified in a previous study as one of ~850 genes expressed in a cell cycle stage-specific pattern, with transcription peaking around the M/G1 transition [32]. To confirm this finding and to investigate the cell-cycle expression patterns for other *ZSCAN5* genes, we synchronized HEK-293 cultures using a double thymidine (TT) block, which synchronizes the cells at early S-phase [33]. We collected cells at different time points after release to test gene expression levels of *ZSCAN5A*, *ZSCAN5B*, and *ZSCAN5D* by qRT-PCR, together with marker genes expressed at specific cell cycle stages [*CCNE1* (expression peak at G1/S), *RRM2* (S), *CDC2* (G2), *BUB1* (G2/M) and *PTTG1* (M/G1)]. *ZSCAN5B* and *ZSCAN5D* showed clear peaks of expression beginning around 14h after the release, consistent with peak transcription during the M/G1 period (Figure 2E; Supplementary Table 1A). *ZSCAN5A* expression showed a similar expression pattern consistent with the published reports although RNA levels peaked somewhat sooner than the M/G1 transition, beginning around G2/M according to our experiments.

#### Mouse and human ZSCAN5B are expressed in populations of actively dividing cells

**Figure 3:**
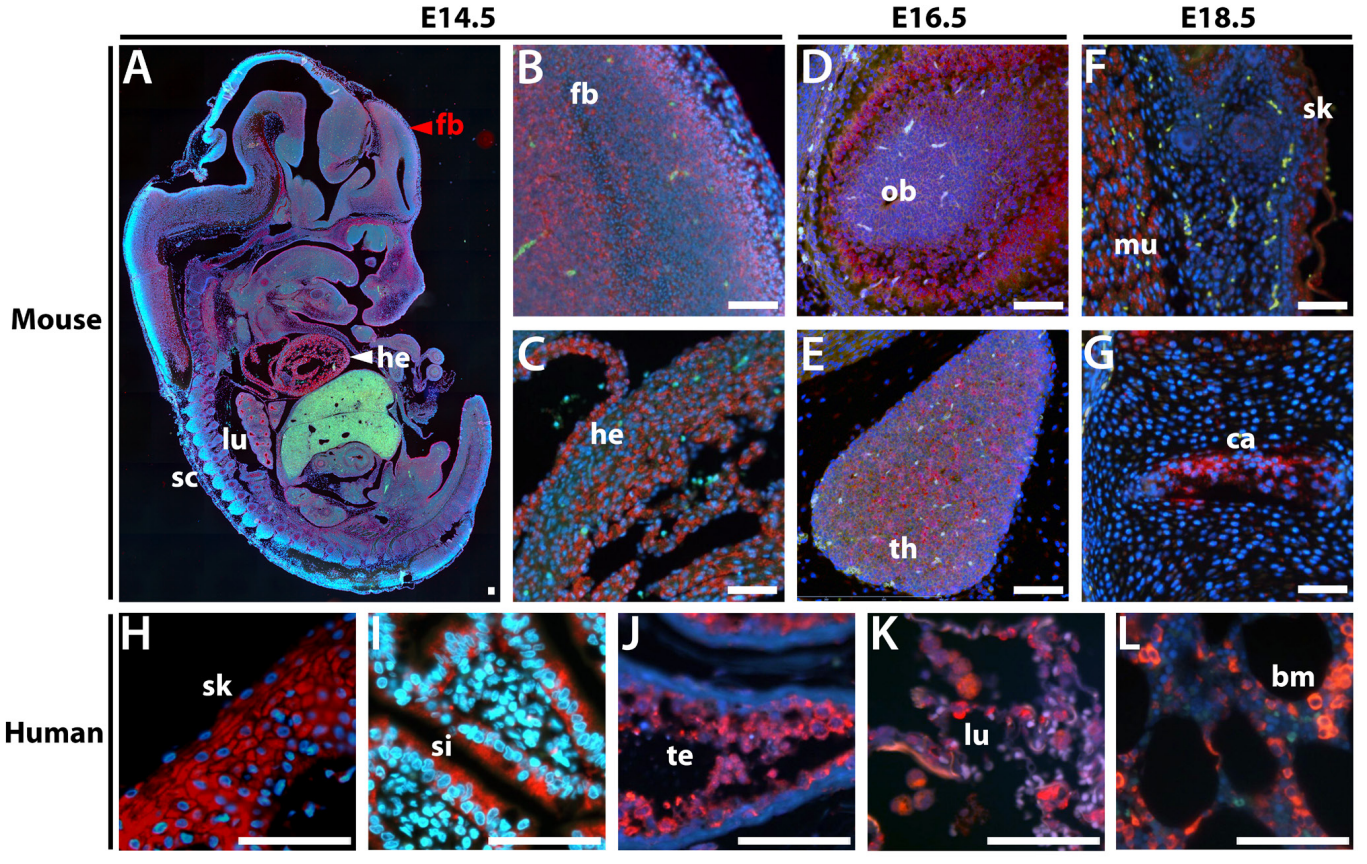
*In situ* hybridization in sectioned embryos and adult human tissues shows that *ZSCAN5B* expression is highest in rapidly dividing cell populations Sagittal sections of paraffin embedded mouse embryos at E14.5, E16.5, and E18.5 were hybridized with a *Zscan5b* antisense RNA probe detected with TSA-Rhodamine (Red), against a DAPI counterstain (blue). **A.** Whole sagittally sectioned E14.5 embryo shows overall tissue expression, with higher magnification panels showing expression in **B.** forebrain (fb), as well as **C.** heart (he). At E16.5, high levels of expression were detected in **D.** olfactory bulb (ob), and **E.** thymus (th). By E18.5, high levels of expression were no longer detected in heart and lung but were predominant in tissues including **F.** muscle (mu), skin (sk), and **G.** cartilage (ca). In human adult tissues, the *ZSCAN5B* gene was detected at particularly high levels in **H.** skin epithelium (sk), **I.** small intestine (si), **J.** testicular spermatocytes (te) and **K.** lung (lu), and bone marrow granulocytes (bm). Scale bar corresponds to 100 μm.

To identify cell type-specific expression patterns for the conserved paralog *in vivo*, we developed *probes* for *in situ* hybridization (ISH) from the unique 3′-untranslated (3′UTR) regions of the mouse and human *Zscan5b/ZSCAN5B* genes. For mouse, we hybridized probes to sagittal sections of whole embryos taken at 14.5 days post-coitum (E14.5), E16.5 and E18.5; for human, we examined paraffin sections of a selection of adult tissues on a tissue array. Mouse *Zscan5b* displayed highest expression in E14.5 heart (Figure 3A, 3C), alveoli of the developing lungs, spinal cord and forebrain (Figure 3A, 3B). Heart and lung expression was diminished but expression remained particularly high in the olfactory bulb (Figure 3D) and thymus (Figure 3E) at E16.5. By E18.5, expression was high in developing skeletal muscle and skin (Figure 3F) and cartilage and lower level in bone in the vertebral column (Figure 3G) the intestinal epithelia; expression detected but at reduced levels in the E18.5 forebrain (not shown). In human tissues, *ZSCAN5B* was also detected in adult skin (Figure 3H), epithelial cells in the small intestine (Figure 3I), testicular spermatocytes (Figure 3J), lung epithelia (Figure 3K), and bone marrow (Figure 3L); thymocytes were also strongly positive for human *ZSCAN5B*, while tissue cores taken from several adult brain regions were not (not shown). In general, therefore, human adult gene expression was highest in tissues and cell types with high levels of cellular turnover and cell division. Interestingly, despite qRT-PCR data suggesting distinct patterns of relative tissue-specific expression levels (Figure 2A), the cell types and tissues that express *Zscan5b* in mouse embryos overlapped considerably with those that displayed high levels of human *ZSCAN5B* in adults.

### Gene knockdown experiments reveal clues to shared and unique cellular functions

Testing RNA from a panel of human cell lines with qRT-PCR confirmed the high and virtually ubiquitous expression of *ZSCAN5A* in cultured cells. However, very few cell lines also expressed *ZSCAN5B* or *ZSCAN5D* (not shown). With the goal of examining paralog function in the same cellular context, we identified two lines in which all three paralogs were expressed - BeWo, a trophoblast-like cell line derived from choriocarcinoma, and HEK-293, derived from human embryonic kidney but with neuronal characteristics [34] for further study.

**Figure 4:**
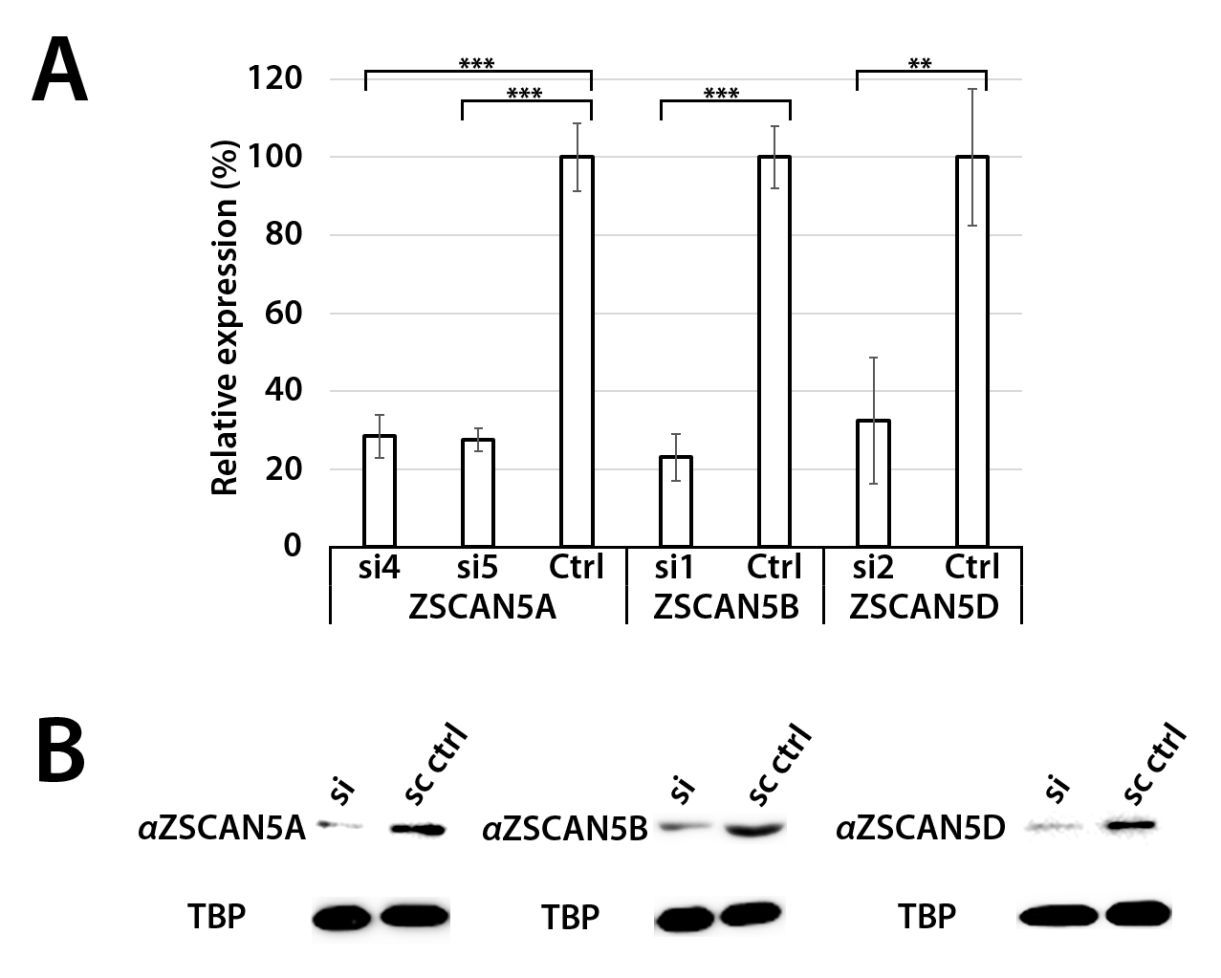
siRNA knockdown of ZSCAN5 and antibody specificity HEK-293 cells were transfected with 10 nM of siRNA targeting ZSCAN5A (si4, si5), ZSCAN5B (si1), or ZSCAN5D (si2) for 48h, after which RNA and nuclear protein extracts were collected. **A.** qRT-PCR of each sample showed 71.6% (si4) and 72.6% (si5) knockdown of *ZSCAN5A* transcripts, 76.9% (si1) knockdown of *ZSCAN5B* transcripts, and 67.6% (si2) knockdown of *ZSCAN5D* transcripts. *P*-values to evaluate the significance of each knockdown were generated from triplicate experiments using one-way ANOVA (**: P ≤ 0.01, ***: P ≤ 0.001) **B.** To assess the knockdown of each protein and to test antibody specificities, Western blots were generated with nuclear protein extracts of the same siRNA knockdowns and stained with antibodies targeting ZSCAN5A, ZSCAN5B, and ZSCAN5D, along with TATA-binding protein (TBP) as an internal control. Western band sizes were detected at 56 kDa (ZSCAN5A, ZSCAN5B, ZSCAN5D), consistent with predicted sizes of those proteins, and 38 kDa (TBP). Relative levels of ZSCAN5 protein to the TBP control in each lane were determined by densitometry, as described in Methods.

Since transfection is particularly efficient for HEK-293 cells, we used HEK-293 for siRNA knockdown experiments. We tested a number of independent siRNA designs, both commercially available and custom, for each gene to assess efficiency and specificity of paralog knockdown. Most siRNA designs displayed off-target effects that significantly reduced levels of at least two of the ZSCAN5 genes under conditions we tested (not shown). However, two of the siRNA reagents (hereafter called si4 and si5) reduced levels of *ZSCAN5A* relatively efficiently. The two siRNA designs differed in their impact on paralogous genes, producing some effect on *ZSCAN5D* (si5) or even some over-expression of *ZSCAN5B* (si4). Additionally, we found a single siRNA design that knocked down *ZSCAN5B* transcripts quite well and specifically (si1). We identified one siRNA design (si2) that allowed a reasonable degree of *ZSCAN5D* knockdown without reducing levels of either of the other two ZSCAN5 genes, but treatment with si2 increased *ZSCAN5B* transcripts levels significantly in HEK-293 cells (Figure 4A; Supplementary Table 1B). Unlike *ZSCAN5A*, we could not find a second siRNA that knocked down *ZSCAN5D* without significantly affecting the levels of the other genes, complicating further functional analysis of this paralog.

We analyzed RNA from HEK-293 cells treated with si1, si4, si5, and a scrambled control using RNA-seq to identify differentially expressed genes (DEGs). Since two independent siRNA designs could be tested for *ZSCAN5A*, gene expression analysis after knockdown of this paralog yielded the most robust and reliable DEG set (363 genes detected with adjusted *P* > 0.05, fold change > 1.5; Supplementary Table 2). Analyzing these DEGs with the DAVID functional analysis program [35] revealed very high enrichment for specific functional categories in the up- and down-regulated genes (Table 1). Notably, genes up-regulated after *ZSCAN5A* knockdown (e.g. negatively regulated by *ZSCAN5A*) included cell cycle regulators especially proteins controlling spindle attachment, chromosome condensation and segregation and the metaphase/anaphase transition. Down-regulated DEGs (positively regulated by *ZSCAN5A*) were significantly enriched for functions including transcriptional regulation and cell-fate commitment in a variety of tissues.

**Table 1:** **Gene Ontology (GO) clusters identified as significantly enriched in gene sets up- or down-regulated after ZSCAN5 gene siRNA knockdown.** (Note: this is new, data from 48h KD)

	ZSCAN5A (DAVID enrichment factor)^1^		ZSCAN5B (DAVID enrichment factor)	
DAVID Functional cluster	Up^2^	Down^3^	Up	Down
Ribonucleotide complex/ribosome biogenesis	9.6			
M-phase/cell cycle	5.0			
Chromosome segregation/mitotic spindle	3.1			
Ubiqutin ligase complex/unfolded protein binding	2.4			
Regulation of metaphase/anaphase transition	2.2			
Condensed chromosome/kinetochore	1.9		1.9	
M-phase of meiotic cell cycle	1.9			
RNA splicing	1.8			
Noncoding RNA metabolic process / tRNA processing			4.7	
tRNA modification/ Wobble uridine modification			2.1	
Mitochondrion			3.4	
Macromolecular complex assembly			2.5	
Transcription regulator activity/regulation of transcription		8.9		3.6
Embryonic morphogenesis		3.2		
Pattern specification process		2.4		
Cell matrix adhesion		2.7		1.9
Extracellular matrix organization		2.2		1.6
Positive regulation of cell migration				2.3
Morphogenesis of a branching structure		2.4		
Tube development/lung development				2.0
Kidney development				1.8
Ectodermal gut morphogenesis		2.5		
Neuron cell fate commitment		2.5		
Axon guidance				1.4
Olfactory bulb development		1.7		
Blood vessel development		2.2		
Cartilage development		1.7		
Hair cycle process/epidermis development		1.7		
Wnt signaling pathway		1.4		
hematopoietic or lymphoid organ development		1.3		

1 David enrichment scores are calculated as the geometric mean of –log transformed P-values of GO terms within a cluster based on content of similar genes

2 see [35]

3 Clusters associated with Up- or Down-regulated genes, respectively.

Genes differentially expressed after human *ZSCAN5B* knockdown displayed significant levels of overlap with *ZSCAN5A* DEGs, with 100 of the 363 *ZSCAN5A* DEGs being detected as similarly up- or down-regulated in the *ZSCAN5B* siRNA experiment (Supplementary Table 2). Since the *ZSCAN5A* knockdown experiments we analyzed did not reduce levels of *ZSCAN5B* and vice versa (Figure 4A), these data suggested some level of functional cooperation between the paralogous proteins. Nevertheless, the *ZSCAN5B* DEG set also emphasized some novel functions including those related to tRNA and rRNA processing and modification (up-regulated DEGs) (Table 1); as for *ZSCAN5A*, genes down-regulated after *ZSCAN5B* knockdown were enriched for functions related to differentiation and development. Interestingly, the tissues predicted to be affected by *ZSCAN5A* and *ZSCAN5B* functions -including kidney, gut, cartilage, hematopoietic/lymphoid tissues, and olfactory bulb - overlapped well with expression sites determined by qRT-PCR and ISH for the human and/or mouse genes (Table 1; Figure 2, Figure 3).

### ZSCAN5 proteins bind to tRNA genes and ETC sites in human and mouse

#### ChIP with paralog-specific antibodies

To identify reagents for detection of ZSCAN5 proteins, we identified commercial antibodies targeting ZSCAN5B and ZSCAN5D, and designed peptide epitopes from a sequence-divergent region of human ZSCAN5A and from the mouse Zscan5b protein to generate custom polyclonal antibodies (see Methods). These antibodies identified nuclear proteins of the correct sizes in BeWo and HEK-293 nuclear extracts which reduced in overall levels after siRNA knockdown of each gene; densitometry revealed a reduction of ZSCAN5A, 5B, and 5D proteins by 84%, 67%, and 80%, respectively (Figure 4B; Supplementary Table 1C). These results confirmed antibody specificities and provided additional support for the functional efficiency of siRNA knockdowns.

We performed ChIP-sequencing using the antibodies to human ZSCAN5A, ZSCAN5B, and ZSCAN5D in HEK-293 and BeWo cells with ChIP-seq from BeWo cells yielding by far the best results. ZSCAN5B peaks in BeWo chromatin were particularly clear, with very little background and strong enrichment in a limited number of genomic positions (a total of 672 peaks; 225 of which were detected with MACS software at a false discovery rate (fdr) = 0; Supplementary Table 2). ZSCAN5A and ZSCAN5D ChIP-seq experiments displayed a higher rate of background but also included larger numbers of clearly enriched peaks. ZSCAN5A and ZSCAN5D ChIP-seq experiments from the HEK-293 cell line were not successful, but ZSCAN5B ChIP yielded a small number of clear peaks (101 peaks) in this cell line. Because antibodies for all three proteins gave excellent results in BeWo chromatin, we focused on results from BeWo ChIP-seq datasets for most types of peak analysis and used the ZSCAN5B HEK-293 ChIP-seq experiments primarily for cell-to-cell comparisons and for functional studies including association of peaks with siRNA knockdown DEGs.

#### ZSCAN5B preferentially binds tDNA in human and mouse cells

We noticed immediately that the summits of the most high-scoring ZSCAN5B peaks in both BeWo and HEK-293 cell lines were positioned centrally inside tRNA genes. In particular, ZSCAN5B peak summits were highly enriched in tDNA sequences (*p* = 0; Table 2). Of the 672 ZSCAN5B BeWo peaks, 240 peaks overlapped with tDNA sequences; 64 of the 74 tDNA peaks identified by ZSCAN5B ChIP-seq in HEK-293 (86.5%) were also identified in this BeWo tDNA peak set (Supplementary Table 2). The ZSCAN5B-bound tDNAs in both cell types correspond to a variety of different amino acids and codons without obvious enrichment of a particular type (Supplementary Tables 2, 3). In all but one case, the bound tDNAs comprised a subset of the 522 loci annotated as active human tRNA genes [36]; a single annotated tDNA pseudogene from chromosome 2 was bound by ZSCAN5B at relatively low efficiency in the BeWo cell line. However, this pseudogene was also identified as bound by both TFIIIC and Pol III in other human cell types, suggesting that it may be expressed ([19]; Supplementary Table 2) Although tRNA expression or Pol III occupancy has not been measured in the BeWo or HEK-293 cell lines, all but 10 of the tDNAs bound by ZSCAN5B in BeWo and all tDNAs bound in HEK-293 cells were found previously to be commonly expressed in a variety of mammalian cell types ([37]; these peaks are marked in Supplementary Table 2). Therefore, we surmise that ZSCAN5B preferentially binds to a subset of the active human tRNA genes.

**Table 2:** Relative enrichment (+) or under-representation (−) of specific repeat families in collections of ZSCAN5, RPC155, TFIIIC, and ETC ChIP peaks

Repeat Family^1^	ZSCAN5B^2^		ZSCAN5A		ZSCAN5D		RPC155^3^		TFIIIC^3^		ETC^3^	
	+/−	*p*-value	+/−	*p*-value	+/−	*p*-value	+/−	*p*-value	+/−	*p*-value	+/−	*p*-value
**tRNA**	+	0	+	0	+	0	+	0	+	0		
**RNA**	+	1.4e-12					+	1.5e-02				
**scRNA**	+	1.9e-08					+	2.9e-19	+	4.3e-02		
**snRNA**	+	4.8e-04					+	5.3e-14				
**Alu**	-	2.2e-08	-	4.3e-25	-	7.9e-70	+	0	+	0	+	0
**L2**	-	2.3e-04			+	7.7e-10	-	1.2e-02	+	4.2e-47	+	6.4e-70
**MIR**	-	3.5e-03			+	1.2e-171	+	4.1e-21	+	3.1e-03	+	5.7e-04
**ERV1**	-	5.0e-03	-	5.0e-04	-	2.5e-14	+	3.3e-07	-	1.8e-10	-	3.7e-06
**telo**			+	7.5e-08								
**Simple_Repeat**			-	4.1e-04			-	1.9e-04	+	1.9e-03		
**srpRNA**			+	2.3e-02			+	1.1e-03				
**Gypsy**			+	2.7e-02					-	4.7e-02	+	3.4e-22
**Low_Complexity**					+	4.4e-23	-	1.4e-06	+	1.5e-50		
**LTR?**					+	4.8e-10						
**Deu**							+	3.1e-02				
**LTR**							+	9.7e-03				
**rRNA**							+	0	+	0	+	1.8e-07
**RTE-BovB**							+	2.2e-03				
**SINE**											+	2.3e-02

1 Repeat family names and locations taken from repeat masker,http://www.repeatmasker.org/; a full accounting with enrichments and depletions for specific elements in each family is provided in Supplementary Table 3.

2 ChIP peaks from the BeWo cell line, focused on all fdr=0 peaks (ZSCAN5B) or all peaks with enrichment > 15 for ZCAN5A and ZSCAN5D, as reported in Supplementary Table 2.

3 Peak coordinates for these feature types are taken from [19], and were lifted over to human genome sequence build hg19 for comparisons.

As a comparison to the human ZSCAN5B ChIP-seq dataset, we used the mouse Zscan5b antibody for ChIP-seq in cells isolated from dissected mouse fetal placenta. We identified 118 peaks, dominated by peaks mapping directly onto a nested subset of the same tDNA sequences, located in syntenically homologous positions, as those detected by the human ZSCAN5B antibody (Supplementary Table 4). These similarities are remarkable, given that the ChIP experiments were used chromatin sources from different species and cellular sources - that is, from transformed human cell lines and mouse fetal placental tissue. Together these data provide strong support for the notion that ZSCAN5B favors binding to a specific subset of conserved tDNAs.

Almost all of the highest-scoring ZSCAN5B and mouse Zscan5b peaks overlapped tDNAs, but ChIP-seq also detected common enrichment in other types of Pol III transcripts including Vault RNA, 7SLRNA, *RMRP*, and U6 snRNA. Accordingly, ZSCAN5B peaks were enriched for many of the same classes of RNA genes that are occupied by Pol III (measured by ChIP-seq with an antibody to the RPC155 subunit) and Pol III TF required for tRNA transcription, TFIIIC, as reported previously by Moqtaderi and colleagues [19] (Table 2). The small number of peaks that did not overlap with RNA Pol III genes was also interesting. For example, several individual peaks with high intensity were found to overlie MIR and Alu SINE repeats, which are evolutionarily derived from tRNA and 7SL RNA, respectively [38]. One particular example, identified in human ZSCAN5B ChIP-seq in BeWo and HEK-293 cells as well as mouse Zscan5b ChIP-seq in fetal placenta corresponds to a MIR repeat located approximately 1 kb downstream of the promoter of *POLR3E*, encoding the RPC5 subunit of Pol III (Supplementary Tables 2, 4); this MIR element has been shown to function as an enhancer for the *POLR3E* gene [17].

#### ZSCAN5A and ZSCAN5D binding sites are also enriched in Pol III transcripts

**Figure 5:**
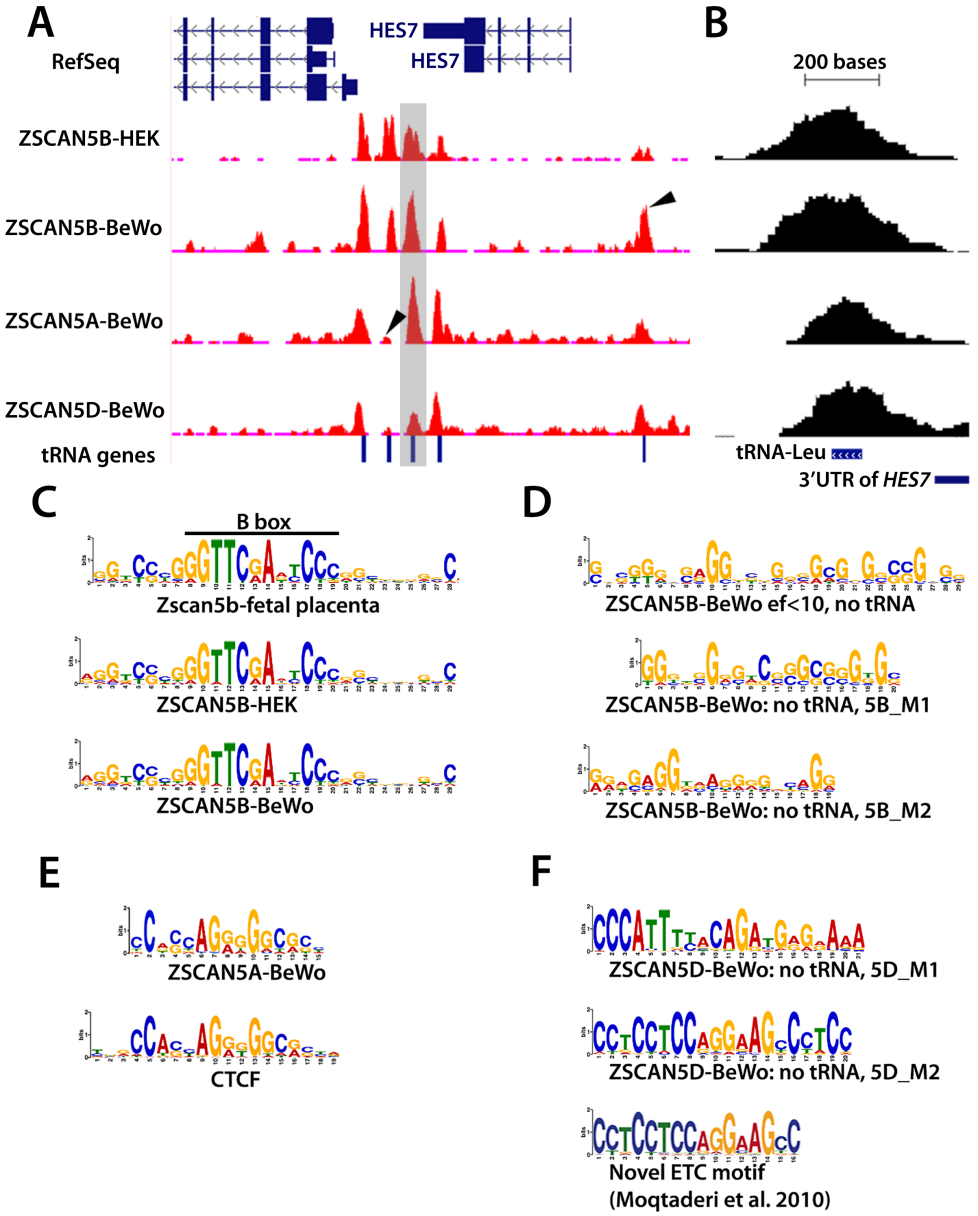
ZSCAN5 binding displays a protein- and cell type-specific preference for tDNAs and other Pol III-related loci enriched in G/C rich motifs **A.** Distribution of ChIP-seq reads in the *HES7* region is displayed in a snapshot from the UCSC browser. ChIP-seq read pileups are shown in red, with the distribution of background reads from genomic input displayed in brown. Peaks from ZSCAN5B in chromatin from two cell lines, HEK-293 and BeWo, shown together with peak profiles from ZSCAN5A and ZSCAN5D ChIP-seq in BeWo chromatin, reveal strong enrichment over tRNA genes that are clustered in the region. Arrows in peak tracks highlight some of the peaks that are differentially enriched by ZSCAN5B ChIP in the two cell types, or in ChIP with ZSCAN5B compared to ZSCAN5A or ZSCAN5D. The tRNA-Leucine (tRNA-L) gene shown in close-up in panel B is highlighted in grey. **B.** A close-up view of the tDNA highlighted in grey in panel A, illustrating that the ChIP-seq peak summits are centered over the tDNAs. **C.** Motifs detected as most highly enriched and central in ChIP-seq peaks for mouse Zscan5b and human ZSCAN5B in HEK-293 or BeWo cells include the TFIIIC-binding B-box, which is present and highly conserved in all expressed tDNAs, and strikingly similar tracts of G/C-rich surrounding DNA. **D.** A motif search conducted after removing tDNA sequences from the ZSCAN5B BeWo peak set also revealed G/C-rich sequences, including an extended motif comprising two shorter motifs (5B_M1, 5B_M2) as predicted ZSCAN5B binding motifs. **E.** A G/C rich central motif was also detected in analysis of ZSCAN5A ChIP peaks; a portion of this motif bears striking resemblance to the known motif for transcription factor and insulator protein, CTCF. **F.** A distinct set of motifs were detected in high-scoring ZSCAN5D peaks, including a G/C rich motif, 5D_M2, which bears striking similarity to a novel ETC motif detected by Moqtaderi and colleagues [19].

ZSCAN5A and ZSCAN5D binding sites were also highly enriched in tDNA sequences (Table 2). In fact, the three proteins appear to co-occupy many tDNA sites or to occupy neighboring tDNAs within the same genomic clusters (Supplementary Table 2) although with different relative efficiencies. One particularly interesting set of examples is illustrated in Figure 5A; the clustered tDNAs in this region are differentially marked by the human ZSCAN5 proteins. A close-up of a tRNA-Leucine (tRNA-L) gene located just downstream of *HES7* and occupied in all of the ChIP experiments (Figure 5B; this tDNA is shaded in Figure 5A) illustrates the general position of the peak summits (or centers of the peaks) relative to the tRNA genes. As shown in Figure 5B, although ChIP peaks were generally broader than the tRNA genes *per se* (due to the standard chromatin shearing size for ChIP, see Methods), the summits are positioned directly over the middle of the genes.

The tDNAs in this chromosome 17 region, which surround the *ALOXE3, HES7* and *PER1* genes, have been demonstrated previously to serve as anchors of local chromatin loops that function as insulators in human cells [10]. tDNAs throughout the genome also displayed ZSCAN5 protein-specific peaks and for ZSCAN5B, cell type-specific enrichment patterns (Supplementary Table 2). These data suggest that the ZSCAN5A, B, and D proteins all bind to tDNA sequences but can have different locus preferences within the same cell type; ZSCAN5B also clearly binds to certain tDNA loci more or less efficiently depending on the cellular context.

### Motif analysis reveals binding preferences for ZSCAN5 proteins

#### ZSCAN5A and ZSCAN5B proteins bind G/C rich motifs

We used the MEME suite [39] to search for enriched sequence motifs enriched at the summits of the highest-scoring ZSCAN5A, ZSCAN5B and ZSCAN5D peak regions (see Methods). The analysis of ZSCAN5B peak summit regions revealed clear enrichment for a sequence including the TFIIIC-binding B box as the top-scoring, centrally located motif; Zscan5b peaks in mouse fetal placenta chromatin also yielded a very similar extended and central B Box motif (Figure 5C). The B Box motif is positioned internally to tRNA genes as an integral component of the Type 2 Pol III promoter [1]; that the B Box is positioned centrally within summits of the collected ChIP peaks indicates that ZSCAN5B binds very near the B Box site. The A box motif typical to Pol III Type 2 promoters was also identified as highly enriched in ZSCAN5B ChIP experiments although the motif was not central to the peaks (not shown). It was not at all surprising to identify these motifs, given that the A and B box elements in tDNAs are very distinct and very well conserved between sites.

However, there are several reasons to doubt that ZSCAN5B protein actually binds to the B Box site. In particular, although tRNA binding sites are by far the most numerous, ZSCAN5B also bound with high affinity to other types of genomic, including regions not associated with Pol III binding and Pol III transcripts - such as U6 and *RMRP* - that are expressed from Type 3 promoters without a B Box motif [1, 40]. However, the enriched motifs also included less distinct G/C-rich DNA sequences extending beyond the B box (Figure 5C), and we therefore hypothesized that ZSCAN5B might in fact bind to this G/C-rich DNA sequence.

To gain more detailed information about the binding motif, we examined 134 highly enriched (MACS enrichment factor or ef > 10) ZSCAN5B BeWo peaks not associated with tDNAs. MEME analysis identified a long (29 bp) G-rich motif located centrally within the peaks (detected at *E* value of 2.6e-26, in 56 of the 134 peak sites). Adjusting MEME parameters to search for shorter motifs (since with 5 zinc fingers, ZSCAN5B is expected to bind at most to a 15 nt target region) identified two similarly G/C-rich motifs, which we call 5B_M1 (detected at *E* value of 6.5e-12) and 5B_M2 (*E =* 1.7 e-02) (Figure 5D). ZSCAN5A peaks were also enriched in B-Box motif (*E =* 6.1e-46) and A-Box motifs (*E =* 5.0e-15), although neither motif was located centrally to the peak regions (not shown). However, MEME analysis identified a G/C-rich motif that was centrally located in ZSCAN5A peak summits. This 5A-enriched motif (detected at *E =* 2.3e-29) bears a striking resemblance to the known binding motif for transcription factor and chromatin organizer, CTCF (Figure 5E). Interestingly, CTCF has been shown to be enriched at mammalian ETC sites [19] and tDNAs [37], and the enrichment of CTCF motifs centrally within ZSCAN5A binding regions suggests that the two proteins may possibly interact or compete at those sites. Whatever this interaction, both ZSCAN5A and ZSCAN5B ChIP-seq experiments identified G/C-rich motifs as potential binding sites.

**Figure 6:**
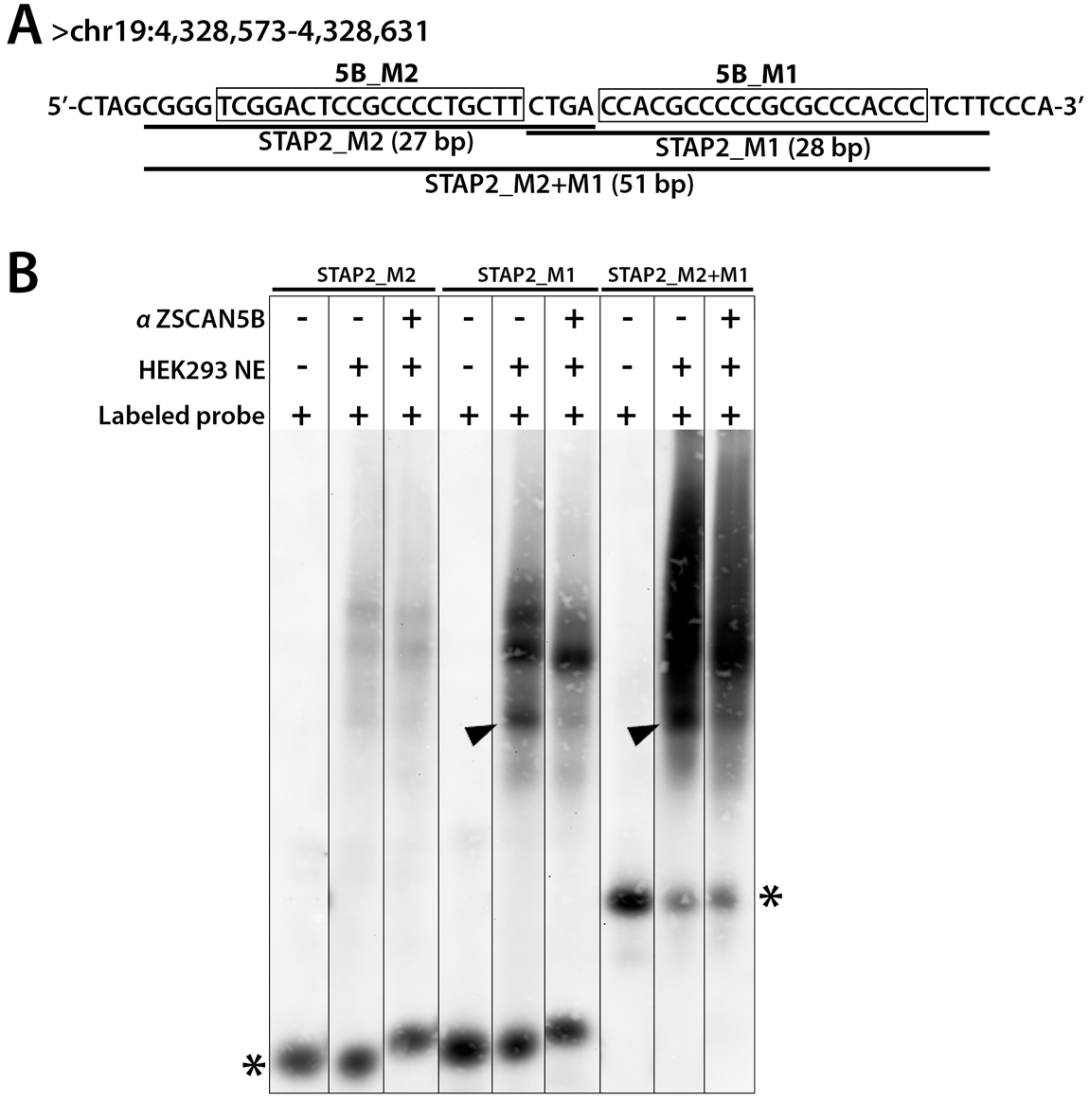
ZSCAN5B proteins bind G/C rich motifs To test the prediction that the ZSCAN5B protein binds to the G-rich motifs, we tested the summit region of a non-tDNA peak uniquely detected with high efficiency by ZSCAN5B in ChIP located within an intron of the STAP2 gene (human assembly GRCh37, chr19:4,328,490-4,328,689) which contains two predicted motifs. **A.** Biotin-labeled oligonucleotides were designed to cover fragments of the peak region including the “5B_M2” motif (boxed) and “5B_M1” (boxed) motif, respectively, and also a longer oligonucleotide spanning both motifs. Labeled probes containing 5B_M2 were named “STAP2_M2” (27 bp), 5B_M1 as “STAP2_M1” (28 bp), and the longer prone as “STAP2_M2+M1” (51 bp). **B.** Nuclear extracts (NE) containing a biotinylated probe (Labeled probe) with or without anti-ZSCAN5B antibody (αZSCAN5B) were resolved on 6% non-denaturing polyacrylamide gels. Addition of αZSCAN5B caused the effective supershift of the lowest band of three “shift” complexes for STAP2_M1 and STAP2_M2+M1 (arrowheads), suggesting that the M1 motif is the preferred binding sequence Asterisks indicate unbound biotinylated DNA probes.

To ask whether the ZSCAN5B protein binds to the G-rich motifs, we selected one highly enriched ZSCAN5B peak region for the “supershift” version of electrophoretic mobility shift assays (EMSA). The tDNAs are repetitive and mostly occupied by the very large Pol III protein complex, and these properties complicate EMSA experiments. Therefore, we focused on a non-tDNA and high intensity peak uniquely detected by ZSCAN5B, located within an intron of the *STAP2* gene (human assembly GRCh37, chr19:4,328,490-4,328,689). The peak summit region contains side-by-side high-scoring matches to 5B_M1 and 5B_M2 motifs. We designed biotin-labeled oligonucleotides that span the two motifs for EMSA testing (Figure 6).

Both double-stranded oligonucleotides from the *STAP2* intronic region were shifted after addition of the HEK-293 nuclear extract, although the 5B_M1 oligonucleotide and a longer oligonucleotide containing both 5B_M1 and 5B_M2 were shifted much more intensely. Addition of the ZSCAN5B antibody caused the smallest of three “shift” complexes for 5B_M1 and the longer oligonucleotide with both motifs (arrowheads in Figure 6) to be supershifted efficiently. Although, given the nature of these experiments, we cannot rule out the possibility that ZSCAN5B might bind these sequences indirectly, e.g. through an intermediate protein also present in the extract, these data indicated that ZSCAN5B binds to the G/C-rich sequence commonly identified in the tRNA and non-tRNA binding peaks.

#### ZSCAN5D binding is enriched at MIR SINEs and LINE2-associated ETC motifs

ZSCAN5D peaks did overlap with ZSCAN5A and ZSCAN5B at tDNAs, but the highest-scoring peaks for the ZSCAN5D antibody were distinctly bound. In fact, a majority (56%) of the ZSCAN5D fdr = 0 peaks were proximal (within 5 kb) to the transcription start site (TSS), while 80% of ZSCAN5A and ZSCAN5B peaks were distal to TSS. Enriched repetitive element classes also distinguished ZSCAN5D peaks from those associated with ZSCAN5A or ZSCAN5B. In particular, unlike ZSCAN5A or ZSCAN5B peaks, ZSCAN5D peaks were enriched for MIR and L2 repeats; in this respect the ZSCAN5D binding regions more closely resembled ETCs (Table 2). Furthermore, analysis of ZSCAN5D binding peaks revealed a distinct set of centrally enriched motifs. The first and most highly enriched motif identified in this peak dataset (5D_M1) corresponded to a novel sequence containing similarity to the known motif for GATA-1 (detected at *E* = 1.2e-524; Figure 5F). The second significantly enriched motif found at the center of predicted ZSCAN5D summits, or 5D_M2 (*E =* 1.4e-293) is notable because it is virtually identical to a novel ETC-associated motif identified previously in human cells [19] (Figure 5F). The two ZSCAN5D peak-enriched motifs were mostly distributed in distinct peak summits, although several ChIP summits contained both predicted motifs in close proximity.

Closer inspection revealed that 5D_M1 mapped frequently within annotated MIR SINE repeats, while 5D_ M2 was contained within LINE2 (L2) elements; both of these repeat types were highly enriched in the ZSCAN5D ChIP peak dataset as well as ETC sites (Table 2). Analysis of peak sequences after masking repetitive elements within them returned centrally located motifs almost identical to 5D_M2 (*E =* 2.2 e-91, not shown) and 5D_M1 (*E =* 1.0e-87). It is therefore likely that ZSCAN5D binds to these motifs whether or not they are embedded in recognizable repeats. These data suggest that ZSCAN5D favors binding to the previously identified ETC motif and that each of the primate-specific ZSCAN5 proteins likely recognize G/C-rich sites that are nevertheless distinct from the sites preferentially bound by parental ZSCAN5B.

### ZSCAN5 protein binding influences expression of bound Pol III and nearby Pol II genes

To ask whether ZSCAN5 protein binding might affect transcription of either the Pol III genes bound by the proteins or the Pol II genes located nearest those sites, we examined ZSCAN5 ChIP-seq peaks that either mapped within or flanked DEGs detected after siRNA knockdown. Since knockdown data could only be reliably supplied by experiments in HEK-293 cells and ChIP-seq was only successful in HEK-293 cells with the ZSCAN5B antibody, we focused primarily on peaks and nearby genes associated with ZSCAN5B in that cell line (Supplementary Table 5).

First, we examined Pol III genes to which the ZSCAN5B protein was actually bound. In the case of tDNAs, the protein sits very centrally over the bodies of the genes, as described above (and illustrated in Figure 5B). The ZSCAN5B peak in *RMRP* is also situated centrally within the body of the gene rather than at its upstream promoter (Supplementary Table 2). ChIP-seq data revealed that *RMRP* was bound by ZSCAN5B in both HEK-293 and BeWo cells and also at lower level by ZSCAN5A in BeWo chromatin (Supplementary Table 2). This gene, encoding the RNA component of RNase MRP, represents one of the very few Pol III genes with a unique sequence composition, permitting accurate measurement of *RMRP* transcript levels in RNA-seq. *RMRP* was significantly up-regulated (by a factor of 3 or 4 fold, respectively) in both *ZSCAN5A* and *ZSCAN5B* siRNA experiments suggesting that ZSCAN5 protein binding suppresses expression of the gene (Supplementary Table 2). Most other Pol III genes are highly repetitive and all, including *RMRP*, display very strong and stable secondary structures, making PCR-based measurement of their expression challenging. However, primer sets that can uniquely detect a small number of tRNAs have been reported [17]. Of the genes expressing these tRNAs (encoding tRNA for tyrosine, or tRNA-Y), one was occupied by ZSCAN5A and ZSCAN5B in BeWo cells and ZSCAN5B in HEK-293; a second gene, encoding to a tRNA for Arginine (tRNA-R), was occupied by ZSCAN5A and ZSCAN5B in BeWo but not bound by ZSCAN5B in HEK-293 cells (Supplementary Table 2). The other tRNA genes for which unique primer sets have been reported were not occupied by ZSCAN5A or ZSCAN5B proteins in either cell type; these primer sets therefore provided excellent negative controls.

We used these validated tRNA primer sets to test expression after ZSCAN5 gene knockdown in HEK-293 cells, along with primers for *RMRP*. Consistent with RNA-seq experiments, *RMRP* was up-regulated after siRNA knockdown as was the tRNA-Y expressed from the locus bound by both ZSCAN5A and ZSCAN5B in HEK-293 cells (Supplementary Figure 1). The tRNA-R expressed by the locus occupied by ZSCAN5A in BeWo but not ZSCAN5B in HEK-293 cells was up-regulated only slightly after *ZSCAN5A* knockdown in HEK-293 cells, although not at significant levels (*p* = 0.09) and was not affected by knockdown of *ZSCAN5B*. Furthermore, the expression of tDNAs not bound by either protein in HEK-293 cells remained unchanged. Although further studies will be required to test the broader generality of this trend, these data support the hypothesis that ZSCAN5 proteins negatively regulate transcription of Pol III genes to which they are bound.

Next we examined expression status of Pol II genes with transcription start sites (TSS) located in the vicinity of ZSCAN5B HEK-293 peaks. ZSCAN5B HEK-293 peaks are almost all located directly on top of Pol III genes, and Pol III genes are not frequently located near Pol II gene promoters. Therefore of the 17 *ZSCAN5B* DEGs identified as nearest to HEK-293 ZSCAN5B ChIP peaks, only eight genes -*HES7, TRIM7, NDUFS7, TIA1, C17ORF59, C16ORF13, GEMIN7, MIR3648 -* were found within 5 kb of the peaks. The first six of the above-mentioned genes are adjacent to peaks overlying tDNAs, and in some cases, clusters of ZSCAN5B-bound tDNAs -for example, *HES7* which is flanked by clustered tDNA peaks (Figure 5A; Supplementary Table 5) - whereas other DEGs are positioned near other types of ZSCAN5B peaks. Of the eight genes with TSS within 5 kb of peaks, only tRNA-linked *TIA1* was down-regulated while all others were up-regulated after *ZSCAN5B* knockdown.

Considering all HEK-293 peak-linked ZSCAN5B DEGs including those with promoters located further from peaks, the picture is more mixed, with 11 genes up-regulated and 6 genes down-regulated after siRNA knockdown (Supplementary Table 5). These gene numbers are too small for concrete conclusions. However, they suggest a trend toward repression of promoters especially those located nearest to ZSCAN5B-occupied peaks. Further, they also hint that the effects of ZSCAN5 protein binding on nearby Pol II genes may not represent direct transcriptional repression or activation, but could involve more complicated mechanisms.

### ZSCAN5A knockdown increases numbers of mitotic cells and aneuploidy

As described above and consistent with previous reports [32], ZSCAN5 paralogs are commonly up-regulated around the time of mitosis (Figure 2). Adding to the intrigue, *ZSCAN5A* knockdown experiments strongly suggested a role for this gene in regulation of the metaphase/anaphase transition (Table 1). To test this hypothesis, we engineered HEK-293 cells to stably carry copies of a plasmid transgene designed to express a tetracycline-regulated (Tet-on) short hairpin RNA (shRNA) based on si4, with the goal of knocking down *ZSCAN5A* controllably and stably in HEK-293 cells.

**Figure 7:**
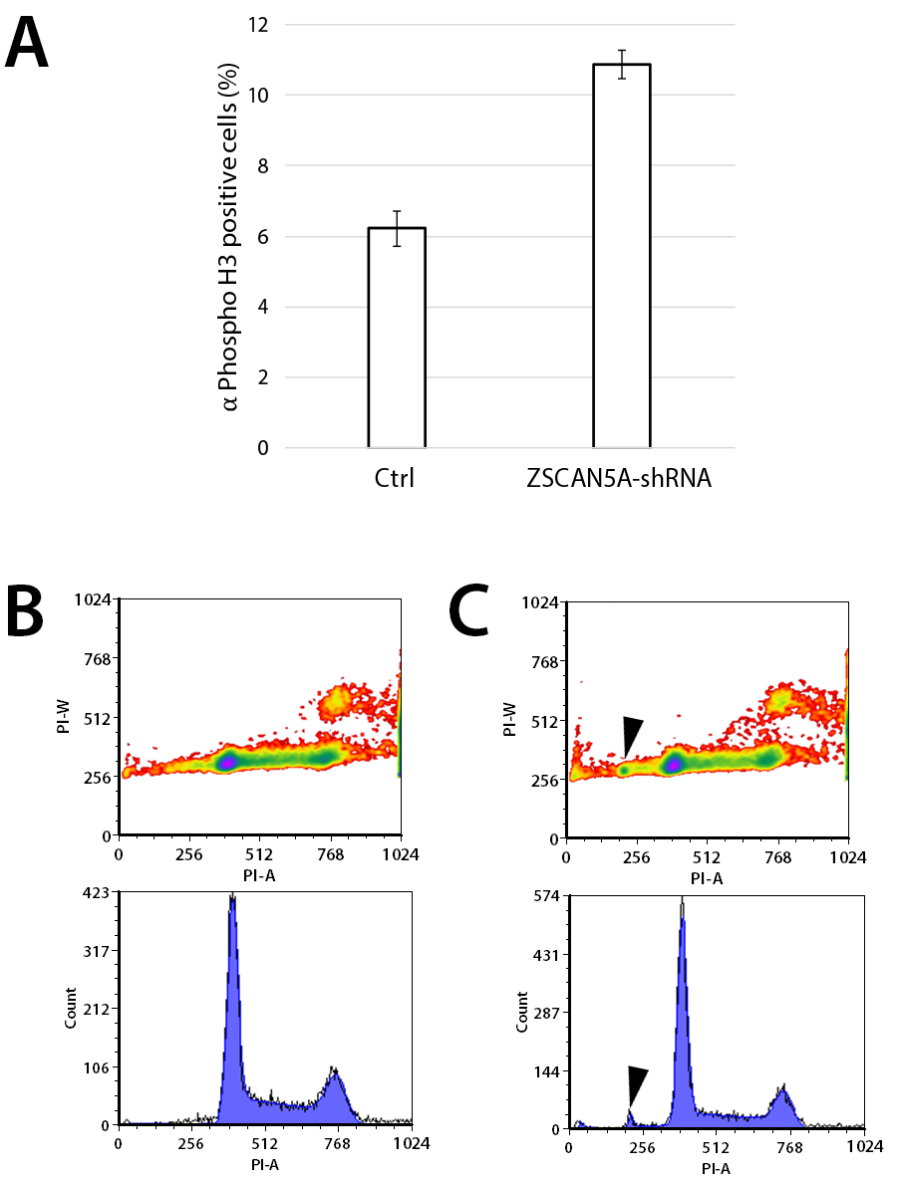
Knockdown of ZSCAN5A increases number of cells entering mitosis and aneuploidy HEK-293 cells engineered to stably carry an inducible short hairpin RNA construct targeting *ZSCAN5A* (ZSCAN5A-Tet-shRNA), or empty-vector control were incubated with 1 μg/mL doxycycline (Dox) for 48h on gelatin-coated glass cover slips. **A.** Fixed coverslips were stained with mitotic marker anti-Phospho-Histone H3 (Ser10) antibody (EMD Millipore), and DAPI to visualize mitotic cells using microscopy, examining totals of more than > 2000 cells per cell line. The number of mitotic nuclei was significantly increased in the shRNA-carrying cells (*p* = 5.04e-12, two-sample test for equality or proportion without continuity correction; see Methods), with standard errors from the counting of multiple samples for each cell type shown as error bars. **B.** Flow cytometry of control and **C.** ZSCAN5A-Tet-shRNA cells taken 48 h after Dox treatment consistently revealed the appearance of a small population of cells (average of 2.01%) with lower DNA content compared to normal G1 cells after ZSCAN5A knockdown (arrows in C). This population was never detected in the Dox-treated (B) or untreated (not shown) control cells we tested. In both experiments, *ZSCAN5A* knockdown rate was determined by qRT-PCR to be 60 % (A) or 43% (B) respectively.

Knockdown of *ZSCAN5A* was confirmed in doxycycline (Dox) treated cells carrying the transgene with qRT-PCR (ranging from 43-53% knockdown in different experiments, not shown). Although this knockdown rate was consistently lower than achieved by siRNA treatment, these cells expressed phenotypes consistent with the functions predicted by the siRNA knockdown experiments. In contrast to cells transfected with an empty plasmid vector, and as evidenced by expression of mitotic marker, phosphorylated Histone H3 (ser10), a significantly larger fraction of *ZSCAN5A* shRNA-expressing cells were detected at the mitotic phase 48 h after addition of Dox, consistent with a less efficient transition to anaphase (Figure 7A; Supplementary Table 1D). Furthermore, flow cytometric analysis revealed the appearance of aneuploid cells after *ZSCAN5A* knockdown, with DNA content lower than that of HEK-293 cells (Figure 7B, 7C). The fraction of aneuploid cells was small in these short-term experiments (around 2%; Figure 7), but highly consistent between experimental replicates and repeat experiments in comparison to Dox-treated vector-only cells, which never yielded this aneuploid population (see Methods). Taken together with the siRNA gene expression results, these data suggest that *ZSCAN5A* depletion leads to abnormalities in spindle assembly or attachment during mitosis, a situation well known to cause metaphase arrest and aneuploidy in mammalian cells [41].

## DISCUSSION

Our study defines the regulatory functions of the ZSCAN5 family of TF proteins for the first time. ZSCAN5 genes are actively expressed in rapidly dividing cell populations and are cell-cycle regulated, with peak expression around the time of the mitosis, and the encoded proteins bind preferentially to Pol III transcription units. ZSCAN5B and primate-specific ZSCAN5A bind preferentially to tDNA, occupying many of the same Pol III genes and similarly affecting the expression of neighboring genes. Based on these data we speculate that the two proteins interact and co-bind DNA as heterodimers through interactions between their nearly identical SCAN dimerization domains.

Our data indicate that when ZSCAN5B binds to Pol III promoters it also acts to negatively modulate the expression of those genes, and suggest that ZSCAN5A has a similar and we conjecture, a cooperative regulatory effect. The mechanisms underlying these functions will require further study but based on data presented here and published reports, we speculate that the differential expression of flanking Pol II transcripts might arise secondarily to dysregulation of the bound Pol III genes.

Indeed, a number of previous studies have pointed to a relationship between Pol III transcriptional activity and the expression of nearby Pol II genes. For example, in budding yeast, actively expressed tDNAs exert repressive effects on nearby Pol II promoters, a phenomenon referred to as “tRNA gene-mediated silencing” [22, 42]. However, similar effects have not been documented in metazoans. Rather, several lines of evidence suggest a more complex relationship in mammals, with tDNAs, MIR repeats, and other active Pol III-transcribed sequences serving enhancer-blocking or barrier-insulating functions [10, 17]. Our data do not argue definitely for a particular model, but do indicate that ZSCAN5B typically represses the activity of Pol III loci to which it is bound, and that the protein may also dysregulate the nearest Pol II genes indirectly through this Pol III binding.

A number of different proteins have been described to regulate Pol III gene transcription in yeasts, in metazoans, or both [3, 43-45]. One deeply conserved Pol III regulator, MAF1, shares many properties with ZSCAN5B and serves as a potential model for its function. From yeast to mammals, MAF1 inhibits transcription from Pol III promoters *via* interactions with TFIIIB [46, 47]. The involvement of TFIIIB is a particularly interesting in this regard, since Type 2 promoters typical of tDNAs and Type 3 Pol III promoters, such as that associated with *RMRP*, share binding of TFIIIB but not TFIIIC or other factors [1]. Of further potential relevance, MAF1 also independently represses Pol II promoters [48], and indirectly represses Pol II promoters located near MAF1 binding sites in Pol III genes. Intriguingly, the indirect repression of neighboring Pol II loci relies on *MAF1-*dependent regulation of Pol III-stimulated chromatin looping. In particular, Lee and colleagues have recently demonstrated that activity of two Pol II genes, *CDKN1A* and *GDF15*, is regulated MAF1 by binding at nearby MIR SINE elements, which inhibits the formation of activating chromatin loops [23].

By analogy, we speculate that ZSCAN5B might also influence chromatin loop formation by negatively modulating activity at bound tDNAs, MIR repeats, and interacting sites; future research will be focused on testing this hypothesis. However, here we should add that chromatin loop formation need not always lead to Pol II gene activation: the remodeling of chromatin contact points can also lead to gene repression through enhancer blocking and other mechanisms (e.g. [8, 24, 49-51]). If our hypothesis regarding ZSCAN5 protein function is correct, the positive or negative effects on Pol II genes could be subtle and far-reaching, extending beyond the nearest promoters and depending on the particulars of interactions within the modulated chromatin loops.

Whether directly or indirectly, *ZSCAN5A* and *ZSCAN5B* knockdown robustly affected the expression of large sets of functionally coherent genes, which together provide important clues to their ultimate biological functions. Consistent with its mitotic expression, DEGs identified after *ZSCAN5A* knockdown strongly predicted functions related to mitotic spindle formation, chromosome segregation and metaphase-anaphase transition; cells engineered to stably knock down ZSCAN5A expression displayed phenotypes that are highly consistent with this prediction. *ZSCAN5B* knockdown further suggested a role in tRNA maturation and modification, and the coordination of these processes with tRNA synthesis makes excellent sense. Here we should note beyond the essential functions tRNAs serve in protein synthesis, tRNA processing also generates fragments that function as microRNAs and serve other independent signaling functions; the regulation of tRNA processing, *per se*, can thus exert wide and profound influences on gene expression and cellular state [13].

Interestingly, many of the biological functions suggested for ZSCAN5 proteins can be encapsulated by a brief summary of known functions for *RMRP*, which is, as we have mentioned, a directly repressed target of ZSCAN5A and ZSCAN5B. *RMRP* has been reported to be essential to the processing of ribosomal RNAs, mitochondrial tRNAs, and microRNA precursors [52] and also processes cellular mRNAs, most notably degrading Cyclin B2 mRNA to permit cell cycle progression at the end of mitosis [53]. *RMRP* mutations are associated with cartilage hair hypoplasia-Anauxetic dysplasia (CHH-AD) spectrum disorders, associated with a range of symptoms including short-limbed dwarfism, skeletal dysplasia, hair abnormalities, immunodeficiency and bone marrow failure, gastrointestinal disorders, cognitive defects, and cancer susceptibility [52, 54]. Rather than being caused simply by loss of RNase MRP function *per se*, many CHH-AD symptoms are thought to reflect malfunction of *RMRP-*derived microRNAs that serve to target essential developmental genes [12]. Disruptions in the tight regulation of this single target locus could thus set off a cascade of events with significant downstream consequences, most of which are consistent with the predicted functions of *ZSCAN5A* and *ZSCAN5B*.

Because we could not knock down *ZSCAN5D* specifically, the biological functions of this primate-specific paralog remain something of a mystery. However, our data show that ZSCAN5D protein displays highest preference for tDNA-derived MIR SINE sites and a subset of LINE2 elements that harbor a previously identified ETC motif [19]. L2 elements have carried the transposition-deficient MIR SINEs as “hitchhikers”, and remnants of the two repeats can often be found closely juxtaposed [55]. The high enrichment of both ZSCAN5D and the human ETCs in MIR SINE and L2 repetitive elements (Table 2), suggests that a subset of ETC sites were distributed as MIR-associated L2 repeats during early mammalian evolution, and that ZSCAN5D has evolved as a preferred regulator of those ETCs distributed sites. Further studies will be required to address these hypotheses definitively.

In closing, we note that despite the fact this TF gene family was elaborated relatively recently the genes have evolved to regulate one of the most ancient sets of essential functions, including tRNA transcription and the fundamental control of three-dimensional chromatin architecture. We surmise that *ZSCAN5B* evolved in early eutherian history to add a novel layer of regulation on a subset of Pol III-transcribed genes, modulating their transcription and their chromatin-organizing functions. The new primate paralogs diverged in fingerprint structure but retained a preference for a similar G-rich binding motif to co-bind with ZSCAN5B at many tDNA sites, likely dimerizing through their nearly identical SCAN domains. Through modulation of Pol III transcription and position effects on neighboring genes, our data suggest that human ZSCAN5A and ZSCAN5B collaborate to control of noncoding RNA processing, cell cycle progression and differentiation in many tissues. Although functional understanding of ZSCAN5D is complicated and still incomplete, the observation that this primate-specific protein binds to MIR-repeat derived ETCs offers a potentially valuable clue to the evolution of mammalian chromatin structure and deserves further investigation.

## MATERIALS AND METHODS

### Ethics statement

This investigation has been conducted in accordance with the ethical standards and according to the Declaration of Helsinki and according to national and international guidelines. All animal work was reviewed and approved by the University of Illinois IACUC committee under protocol number 15425.

### RNA preparation and quantitative RT-PCR

Total RNA was isolated from cell lines and tissues using TRIzol (Invitrogen) followed by 30 min of RNase-free DNaseI treatment (NEB) at 37°C and RNA Clean & Concentrator^TM^-5 (Zymo Research). 2 μg of total RNA was used to generate cDNA using Superscript III Reverse Transcriptase (Invitrogen) with random hexamers (Invitrogen) according to manufacturer's instructions.

Resulting cDNAs were analyzed of transcript-specific expression through quantitative reverse-transcript PCR (qRT-PCR) using Power SYBR Green PCR master mix (Applied Biosystems) with custom-designed primer sets (Supplementary Table 6) purchased from Integrated DNA Technology. Relative expression was determined by normalizing the expression of all genes of interest to either human or mouse Tyrosine 3-monooxygenase/tryptophan 5-monooxygenase activation protein, zeta polypeptide (*YWHAZ*) expression (ΔCt) as described [56].

### *In situ* hybridization

Mouse embryos were collected, paraffin embedded, sectioned and hybridized with an antisense RNA probe, essentially as previously described [57]. We generated *In Situ* hybridization (ISH) probes correspond to nucleotides of the mouse *Zscan5b* cDNA sequence (NM_133204), and to human ZSCAN5B (NM_001080456) (See Supplementary Table 6 for probe sequences). Probes were cloned into the pGEM-T vector (Promega) and sequence-validated before being used for ISH. The reverse primer included a T7 promoter sequence to permit antisense RNA generation using the Roche DIG RNA Labeling kit (SP6/T7) (Roche Applied Science) according to manufacturer's instructions. To prepare human tissue arrays, paraffin blocks containing formalin-fixed tissues from normal anonymous adult donors were purchased from NoblePath Inc. Tissue microarray (TMA) blocks were generated using a Tissue Arrayer (Beecher Instruments). The pre-cut paraffin tissue sections were checked by H&E staining for tissue index selection. Forty-one 1.5mm diameter cores were included in the arrays, with 2 cores included to represent different regions of some tissues (x2). High quality 4-micron sections were generated using a Leica ST 2155 microtome. Slides were baked at 41^°^C overnight and stored at −20^°^C until use. Slides were pretreated and hybridizations were performed as described previously [58]. Sections were mounted using Vectashield Mounting Medium with DAPI. Fluorescent images were reviewed using an Olympus BX60 microscope and captured by an Olympus CC-12 digital camera.

### Cell culture and cell cycle synchronization

HEK-293 (ATCC, CRL-1573), Neuro-2a (ATCC, CCL-131), and BeWo (ATCC, CCL-98) cell lines were obtained from the American Type Culture Collection. HEK-293 and Neuro-2a cells were maintained in Dulbecco's Modified Eagle's Medium (DMEM) containing 2 mM L-glutamine, 10% fetal bovine serum (FBS), 1X Pen Strep, and BeWo cells in DMEM/F12K containing 2 mM L-glutamine, 10% FBS, 1X NEAA, 1X Pen Strep, incubated at 37^°^C in 5% CO2. For HEK-293 cell cycle synchronization, 2 mM thymidine was added to HEK-293 cells grown to about 30% confluency and the cells were subsequently incubated for 18h. Thymidine was removed by washing with 1X PBS three times, and adding fresh media, followed by a further 9h-incubation. The second round of 2 mM thymidine was then added and cells were incubated for an additional 15h. Cells were released from G1/S to S by washing with 1X PBS and adding fresh media, and were collected at different time points thereafter.

### Plasmids and transfections

For siRNA knockdown, approximately 4.5×10^5^ HEK-293 cells were seeded to 6-well plates 24h before transfection. Cells were treated with 10 nM of siRNA specific to each ZSCAN5A (si4: SI00779436, si5: SI04221826, Qiagen) or ZSCAN5B (si1: SI00503300, Qiagen), or ZSCAN5D (si2: SI02804774, Qiagen) with a scrambled negative control (Silencer negative control No.1 siRNA, Ambion) for 48h using Lipofectamine RNAi MAX transfection reagent (Invitrogen) according to manufacturer's instructions.

To create a cell line that expresses an inducible short hairpin RNA (shRNA) targeting ZSCAN5A, an annealed double stranded oligonucleotide including to the si4 siRNA sequence was cloned into the pSuperior. Puro plasmid (Oligoengine). The resulting ZSCAN5A-Tet-shRNA plasmid was sequenced before subsequent transfection. For plasmid DNA transfection, about 4.5×10^5^ HEK-293 cells were seeded to 6-well plates 24h before, and 3 μg of ZSCAN5A-Tet-shRNA or empty plasmid was transfected using Lipofectamine 2000 (Invitrogen). 24h later, transfected cells were selected under 1 μg/ml of puromycin for additional 14 days. Single colonies were selected and expanded, then tested for the efficiency of ZSCAN5A by qRT-PCR knockdown 48h after addition of 1 μg/mL doxycycline (Dox; Sigma-Aldrich). Each colony was analyzed of its knockdown efficiency of transcripts and proteins compared to Dox-treated cells carrying the empty vector.

### RNA-seq and computational analysis

48h after siRNA treatment, total RNA was prepared and tested for quality using an Agilent BioAnalyzer and Illumina libraries generated using the KAPA Stranded mRNA-Seq kit with mRNA Capture Beads (Kapa Biosystems, KK8420). Sequencing was performed on an Illumina Hi-Seq 2000 instrument at the University of Illinois Roy J. Carver Biotechnology sequencing facility, to yield 60-65 million reads per sample. All sequencing data described in this paper have been submitted to the Gene Expression Omnibus database (accession number GSE85045).

RNA-seq data were analyzed using the Tophat-Cufflinks Suite of tools [59]. For *ZSCAN5A* knockdown, expression results from si4 and si5 were analyzed as a group in comparison with the scrambled control. Genes identified as differentially expressed with *p* < 0.05 (after Benjamini-Hochberg correction for multiple testing) compared to the negative control-treated samples were considered for further analysis. For *ZSCAN5B* knockdown, which was effective only for a single siRNA design, we considered all genes with expression levels of at least 1 FPKM in at least one sample and considered genes with > 1.5 X fold change relative to scrambled control as DEGs. siRNA up-regulated and down-regulated genes were analyzed for function separately using the DAVID [35] functional clustering algorithm with default settings.

### Protein preparation, western blots, and antibodies

Nuclear Extracts were prepared with NucBuster^TM^ Protein Extraction Kit (Novagen) and measured by Bradford-based assay (BioRad). The extracts were stored at −80^°^C and thawed on ice with the addition of protease inhibitor Cocktail (Roche) directly before use. 15 μg of nuclear extracts were run on 10% acrylamide gels and transferred to hydrophobic polyvinylidene difluoride (PVDF) membrane (GE-Amersham, 0.45 μm) using BioRad Semi-dry system, then visualized by exposure to MyECL Imager (Thermo Scientific).

Rabbit polyclonal antibodies were generated by injection of synthetic peptides corresponding to the tether regions of ZSCAN5A (DLVRAKEGKDPPKIAS) and mouse Zscan5b proteins (CPEPANPQPEKQVDSL); peptide synthesis, antibody production, and affinity purification of antibodies against the purified peptide epitope we carried out by Abgent Inc. ZSCAN5B (sc-249845, Santa Cruz Biotechnology) and ZSCAN5D (ARP47809_P050, Aviva Systems Biology) antibodies were obtained from commercial sources. Antibody preparations were tested for protein specificity and efficiency by Western blot staining along with anti-TATA binding protein control antibody (1TBP18, Abcam),

### Immunocytochemistry and m-phase counting

Stably transfected cell lines were maintained in a regular medium described above with the addition of 0.5 μg/ml puromycin. For immunocytochemistry (ICC), each cell was seeded and grown on gelatin-coated cover slips for 24h using the condition described above with or without 1 μg/ml of Dox. After washing with 1X PBS, cells were fixed with −20^°^C methanol and incubated at −20^°^C for 30 mins and wash three time with 1X PBS again. Blocking was done by incubating the coverslips in antibody diluent (Dako) at 4^°^C for 2h. Primary antibodies targeting ZSCAN5A (1:2000 dilution), ZSCAN5B (1:2000 dilution), and ZSCAN5D (1:2000 dilution), described above; anti-Gamma-tubulin (Abcam, 1:5000 dilution), and anti-Phospho-Histone H3 (Ser10) (EMD Millipore, 1:5000 dilution) were also used. Secondary antibodies targeting primary antibodies were either conjugated with Alexa Fluor^®^ 568 (1:5000 dilution) or Alexa Fluor^®^ 488 (1:5000 dilution) and purchased from Thermo Scientific. Mounting was done using ProLong^®^ Gold Antifade Mountant with DAPI (Thermo Scientific) and confocal microscopy imaging was done using confocal microscope Zeiss LSM880 in the Institute for Genomic Biology Core Facility at the University of Illinois at Urbana-Champaign. Ten samples, totaling more than 2000 cells, were processed from each cell type and total and mitotic cells were counted using Image J software [60]. To determine if the difference in ratios of mitotic cells between two conditions was significant we used the R function prop.test from the R stats library, which uses Pearson's chi-squared test to calculate a p-value. For each pair-wise test the count of mitotic cells and the count of the total cells was passed to prop.test for the two conditions being tested. The prop.test parameter “correct” (Yates' continuity correction) was set to false. Values are summarized in Supplementary Table 1D. To calculate individual 95% confidence intervals for the ratio of mitotic cells in each condition we used the R function binom.test from the R stats library using default settings. These confidence intervals were used to display error bars in Supplementary Table 1D.

### Flow cytometry

In order to measure the effects of ZSCAN5 on G1/S/G2 distribution, flow cytometry was done using above described transgenic cell lines stained with propidium iodide. Cells were grown as described above with 1 μg/mL Dox for 48h. Each sample was trypsinized using 0.25% trypsine/EDTA (Gibco) and washed with 1X PBS with 0.2% FBS. Cell pellets were fixed with cold ethanol and stored at −20^°^C for 24h. Fixed cells were washed twice with 1X PBS and stained with FxCycle™ PI/RNase Staining Solution (Thermo Scientific) at room temperature for 30 min. The BD LSRII Flow cytometry analyzer was used at the Flow Cytometry facility at the University of Illinois at Urbana-Champaign and resulting data were processed and analyzed using FCS Express 5 software.

### Chromatin immunoprecipitation

Chromatin immunoprecipitation was carried out as essentially as described [61] with modifications for ChIP-seq. Chromatin was prepared from HEK-293, BeWo, and Neuro-2a cell lines. About 1.0 × 10^6^ Cells were fixed in PBS with 1% formaldehyde for 10 min. Fixing reaction was stopped with addition of Glycine to 0.125M. Fixed cells were washed 3x with PBS+Protease inhibitor cocktail (PIC, Roche) to remove formaldehyde. Washed cells were lysed to nuclei with lysis solution - 50 mM Tris-HCl (pH 8.0), 2 mM EDTA, 0.1% v/v NP-40, 10% v/v glycerol, and PIC - for 30 min on ice. Cell debris was washed away with PBS with PIC. Nuclei were pelleted and flash-frozen on dry ice.

To obtain chromatin samples from mouse fetal placenta, pregnancies were identified in C57BL/6J females by detection vaginal plugs (day of plug detection is E0.5), and placentas were dissected at E17.5. After removing maternal decidua, samples were homogenized in cold PBS containing PIC using a loose-fitting Dounce homogenizer. Debris was removed using cell strainer (Fisher Scientific), and single cell suspension was washed twice with cold PBS. Viable cells were counted using hemocytometer and crosslinked with 4% paraformaldehyde (Electron Microscopy Sciences) for 10 min at room temperature before nuclear isolation. Cross-linked chromatin was prepared and sonicated using Bioruptor UCD-200 in ice water bath to generate DNA fragments 200-300 bp in size. Twenty micrograms of each antibody preparation, or 20 μg IgG for mock pulldown controls, were incubated with chromatin prepared from nuclei of approximately 5 million cells.

DNA was released and quantitated using Qubit 2.0 (Life Technologies) with dsDNA HS Assay kit (Life Technologies, Q32854), and 15 ng of DNA was used to generate libraries for Illumina sequencing. ChIP-seq libraries were generated using KAPA LTP Library Preparation Kits (Kapa Biosystems, KK8232) to yield two independent ChIP replicates for each antibody. We also generated libraries from sonicated genomic input DNA from the same chromatin preparations as controls. Libraries were bar-coded with Bioo Scientific index adapters and sequenced to generate 15-23 million reads per duplicate sample using the Illumina Hi-Seq 2000 instrument at the University of Illinois W.M. Keck Center for Comparative and Functional Genomics according to manufacturer's instructions.

### ChIP-Seq data analysis

Human ZSCAN5A, ZSCAN5B, and ZSCAN5D ChIP-enriched sequences as well as reads from the input genomic DNA were mapped to the HG19 human genome build, and Mouse Zscan5b ChIP reads and input mapped to the mouse Mm9 genome assembly, using Bowtie 2 software [62] allowing 1 mismatch per read but otherwise using default settings. Bowtie files were used to identify peaks using MACS software (version 14.2) [63], with default settings. All samples, including the Mouse Zscan5b and human ZSCAN5B ChIP samples isolated from HEK-293 cells, both of which had higher levels of background compared to other samples generated, were also analyzed using the more sensitive HOMER software package (http://homer.salk.edu/homer/ngs/index.html) using default conditions for the TF setting and false discovery rate cutoffs of 0.1 (mouse Zscan5b ChIP) or 0.01 (for ChIP in human HEK-293 and BeWo chromatin). After comparison of the individual files, sequence reads from the two separate ChIP libraries were pooled and a final peak set determined in comparison to genomic-input controls. Peaks were mapped relative to nearest transcription start sites using the GREAT program [64]. Peak locations reported by Moqtaderi *et al.* [19] and Oler *et al.* [37] were converted to Hg19 coordinates using the Liftover utility provided by the UCSC genome browser (https://genome.ucsc.edu/), and overlaps between these peaks and ZSCAN5 ChIP data were determined. DEGs and ChIP peaks, with overlaps to these other datasets and repeat-sequence mapping are summarized in Table S2 for human cell types and Table S3 for mouse fetal placenta ChIP.

### Repetitive element overlap analysis

To identify enrichment or under-representation of repetitive element types or families in the ChIP-peak datasets, we used a method modified from that described by Cuddapah and colleagues [65]. Human repeat data were retrieved on 11/25/2013 as the RepeatMasker Table (www.repeatmasker.org) from USCS's table browser (genome.ucsc.edu) [66] with the following parameters: assembly = ‘Feb. 2009 (GRCh37/hg19)’, group = ‘Variation and Repeats’, track = ‘RepeatMasker’, table = ‘rmsk’, region = ‘genome’, output format = ‘BED - Browser extensible data’. The human chromosome sizes required for the analysis were retrieved on 2/20/2014 from the hg19.chromInfo table of the UCSC public database [67]. We examined overlap between genome coordinates of repeat element features and 100 bp intervals surrounding the summits of peaks determined by MACS software from ZSCAN5A, ZSCAN5B, and ZSCAN5D ChIP experiments (986 peaks with FDR = 0 or effective fold change ≥ 15 for ZSCAN5A_BeWo_ef15; 225 peaks with FDR = 0 for ZSCAN5B_BeWo; 1885 peaks with FDR = 0 for ZSCAN5D_BeWo, and all 102 peaks reported by MACS for ZSCAN5B_HEK-293) using the BEDTools intersect function [68]. Overlaps were also determined for peak sets from Moqtaderi and colleagues [19] after applying the UCSC liftover ultility to identify coordinates in the human hg19 genome build (ETC with 1865 200 bp peaks; RPC155 with 1518 200 bp peaks; and TFIIIC with 5472 200 bp peaks).

For each peak set 500 random sets of the same number and peak size were generated by the BEDTools random function, and overlaps between these random peak sets and repeats were counted for each of the 500 random sets. For each repeat element and family, the average overlap count of the random sets and the standard deviation was determined. Then for each repeat element and family a Z-score was calculated using the overlap count of the peak set, and the average overlap count and standard deviation of the random sets. If the overlap count of the peak set was less than or equal to the average of the random sets z was calculated as: $z=\frac{\left(overlap\;count\;of\;peak\;set\right)-\left(average\;overlap\;count\;of\;random\;sets\right)}{standard\;deviation\;of\;the\;overlap\;count\;of\;random\;sets}$. If the overlap count of the peak set was greater: $z=\frac{\left(average\;overlap\;count\;of\;random\;sets\right)-\left(overlap\;count\;of\;peak\;set\right)}{standard\;deviation\;of\;the\;overlap\;count\;of\;random\;sets}$. The R function pnorm(z) was used to calculate a p-value to indicate if the overlap count was significantly under-represented or enriched in a ChIP-peak set when compared to the overlap counts of the random sets. Repeat families or specific elements that were significantly enriched in at least one of the ChIP peak sets are reported in Table 2 and Supplementary Table 3, respectively, along with p-values determined for enrichments or under-representation of that family or element type in each peak set.

### Motif analysis

To identify enriched motifs, we used sequence from a 200 bp region surrounding the predicted summits of selected peaks for analysis with MEME-ChIP with default parameters (Machanick and Bailey, 2011). Motifs displayed in Figure 5 were identified from peaks with the following cutoffs: (panel C) All HOMER-derived peaks for mouse Zscan5b ChIP in fetal placenta chromatin; all HOMER peaks with MACS enrichment factor (ef) > 20 in human ZSCAN5B ChIP with HEK-293 chromatin; and All MACS ef > 20, fdr = 0 peaks from ZSCAN5B ChIP in BeWo chromatin; (panel D) all peaks *except* those overlying tDNAs, identified with MACS in BeWo chromatin with ef > 10; (panels E and F) ZSCAN5A and ZSCAN5D peaks identified with MACS at ef > 20, respectively.

### Supershift electrophoretic mobility shift assay

Nuclear extracts from subconfluent HEK-293 cells were prepared with NucBuster^TM^ Protein Extraction Kit (Novagen) and measured by Bradford-based assay (BioRad). Probes were synthesized as double stranded oligonucleotides by annealing 2 μg of biotin 5′-end labeled single stranded oligonucleotides and unlabeled complementary single stranded oligonucleotides (Integrated DNA Technology) in annealing buffer (10 mM Tris-HCl, pH7.5, 50 mM NaCl, 1 mM EDTA). Mixed oligonucleotides were heated in 95^°^C hot block for 10 min and slowly cooled down to room temperature. The sequences of the probes used were as follows: STAP2_M2 forward (5′biotin- CGGGTCGGACTCCGCCCCTGCTTCTGA-3′) and reverse (5′-TCAGAAGCAGGGGCGGAGTCCGACCCG-3′); STAP2_M1 forward (5′biotin-CTGACCACGCCCCCGCGCCCACCCTCTT-3′) and reverse (5′-AAGAGGGTGGGCGCGGGGGCGTGGTCAG-3′); STAP2_M2+M1 forward (5′biotin-CGGGTCGGACTCCGCCCCTGCTTCTGACCACGCCCCCGCGCCCACCCTCTT-3′) and reverse(5′-AAGAGGGTGGGCGCGGGGGCGTGG TCAGAAGCAGGGGCGGAGTCCGACCCG-3′). EMSA binding reactions were performed at room temperature for 30 min and consisted of 3 μg of nuclear extract in 1X binding buffer, 2.5% glycerol, 5 mM MgCl_2_, 50 ng/μl poly(dI-dC), 0.05% NP-40, and 20 ng of biotinylated DNA probes. For supershift reactions, 5 μg of antibody targeting ZSCAN5B was carefully added and mixed, and incubated for additional 30 min at room temperature. The mixture was run on 6% non-denaturing polyacrylamide gels in 1X Tris borate-EDTA buffer. Protein-DNA complexes were then transferred to PVDF membrane using the BioRad Semi-dry system and cross-linked using the Spectrolinker XL-1000 UC cross-linker (Spectronics Corp.). Detection of biotin-labeled DNA was performed using the LightShift chemiluminescent EMSA kit (Thermo Scientific) and visualized by exposure to MyECL Imager, a charge-coupled device camera (Thermo Scientific).

## SUPPLEMENTARY MATERIALS FIGURE AND TABLES














