# Supplementary material for: ZSCAN5B and primate-specific paralogs bind RNA polymerase III genes and extra-TFIIIC (ETC) sites to modulate mitotic progression
Source: Oncotarget. 2016 Oct 6;7(45):72571–92. doi: 10.18632/oncotarget.12508 (PMC5340127; doi:10.18632/oncotarget.12508)
Supplement: Supplementary file 1 [file oncotarget-07-72571-s001.pdf]

ZSCAN5B and primate-specific paralogs bind RNA polymerase III genes and extra-TFIIIC (ETC) sites to modulate mitotic progression

Supplementary Material

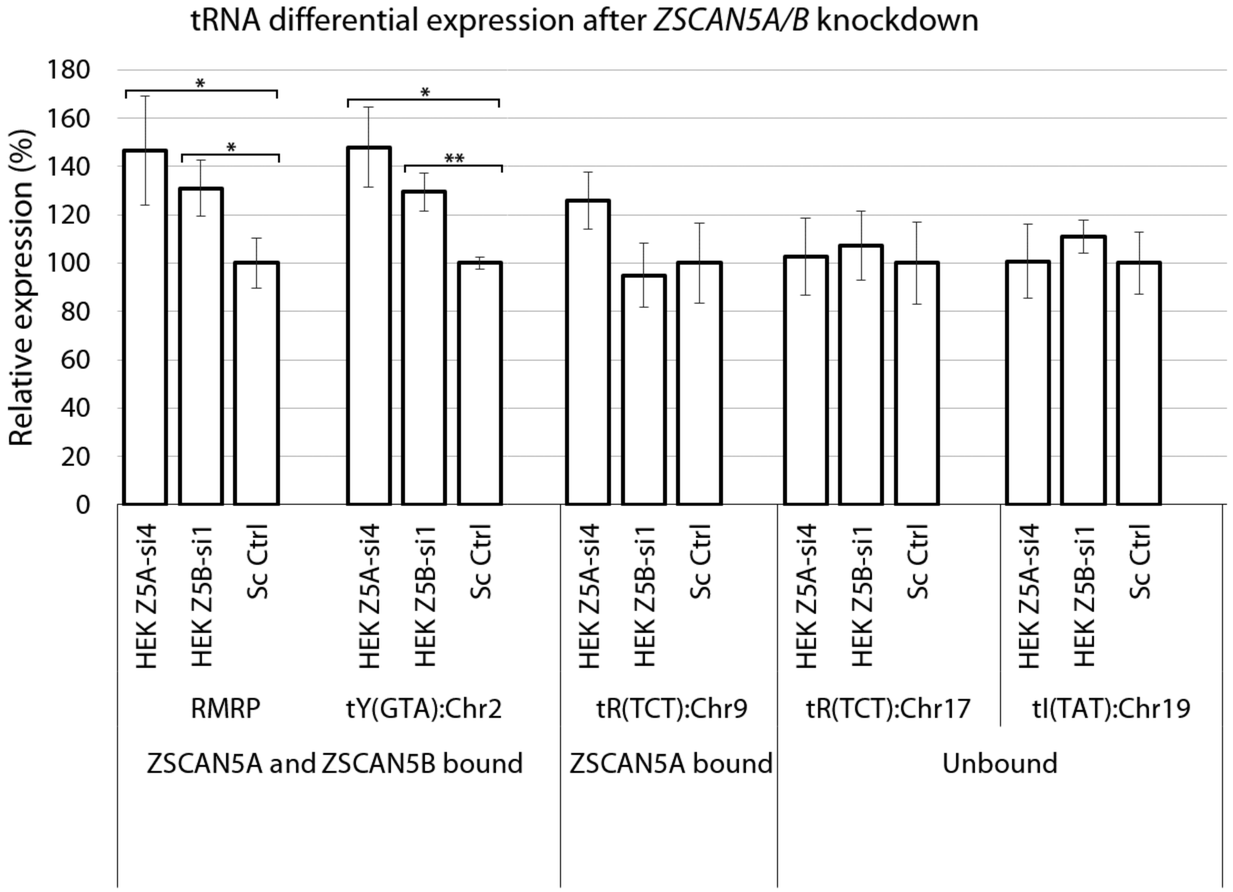

Supplementary Figure 1: Differential expression of ZSCAN5-bound RMRP and tRNAs

qRT-PCR was done to measure the differential expression of gene loci that are bound and unbound by ZSCAN5A and ZSCAN5B after ablation of each gene by siRNA treatments in HEK-293 cells. Error bars correspond to the standard deviations between experimental triplicates. *P*-values to evaluate the significance of the expression differences were calculated from triplicate experiments using one-way ANOVA (*:$P \leq 0.05$, **: $P \leq 0.01$).
