# Supplementary material for: ZSCAN5B and primate-specific paralogs bind RNA polymerase III genes and extra-TFIIIC (ETC) sites to modulate mitotic progression
Source: Oncotarget. 2016 Oct 6;7(45):72571–92. doi: 10.18632/oncotarget.12508 (PMC5340127; doi:10.18632/oncotarget.12508)
Supplement: Supplementary file 2 [file oncotarget-07-72571-s002.docx]

**Supplementary Table 1A: Cell cycle stage-specific expression of ZSCAN5 family genes**

Heat map was created based on the expression value (table) acquired from qRT-PCR of time-series sample collections of HEK-293 cells after the release from double thymidine blocks (see methods). Expression levels of *CCNE1*, *RRM2*, *CDC2*, *BUB1* and *PTTG1* were measured on the same cDNA samples to monitor progression through the cell cycle.

|  | 2h | 6h | 10h | 14h | 18h | 22h |
| --- | --- | --- | --- | --- | --- | --- |
| ZSCAN5A | 475.65 | 490.855 | 723.315 | 695.06 | 690.885 | 374.59 |
| ZSCAN5B | 25.595 | 34.48 | 36.125 | 59.69 | 41.675 | 22.26 |
| ZSCAN5C | 8.875 | 18.62 | 12 | 18.025 | 22.675 | 9.21 |
| ZSCAN5D | 99.605 | 106.95 | 118.43 | 151.965 | 108.4 | 71.235 |
| CCNE1 | 6762.175 | 5984.81 | 4737.2 | 5808.575 | 5270.755 | 7847.29 |
| RRM2 | 39861.63 | 64851.08 | 36734.89 | 32082.56 | 34015.94 | 29267.47 |
| CDC2 | 5326.05 | 7918.475 | 13412.69 | 13568.86 | 6124.265 | 6173.055 |
| BUB1 | 16037.34 | 25008.09 | 38027.1 | 57032.77 | 23263.43 | 13312.39 |
| PTTG1 | 10868.12 | 15055.15 | 23568.57 | 30041.94 | 30999.69 | 16495.17 |

**Supplementary Table 1B: Analyses of affected ZSCAN5 family transcript expression level after specific ZSCAN5 gene ablation**

We used qRT-PCR to measure the level of cross-reactivity among ZSCAN5 family after individual siRNA treatments (see Methods). Standard deviations were measured from experimental triplicates.

|  | ZSCAN5A  (%) | STDEV |  |  | ZSCAN5B  (%) | STDEV |  |  | ZSCAN5D  (%) | STDEV |
| --- | --- | --- | --- | --- | --- | --- | --- | --- | --- | --- |
| HEK ZSCAN5A si4 | 28.4 | 5.5 |  | HEK ZSCAN5A si4 | 148.7 | 13.4 |  | HEK ZSCAN5A si4 | 59.0 | 9.1 |
| HEK ZSCAN5A si5 | 27.4 | 3.0 |  | HEK ZSCAN5A si5 | 95.3 | 29.8 |  | HEK ZSCAN5A si5 | 46.4 | 4.6 |
| HEK ZSCAN5B si1 | 106.2 | 3.9 |  | HEK ZSCAN5B si1 | 23.1 | 6.0 |  | HEK ZSCAN5B si1 | 73.7 | 13.4 |
| HEK ZSCAN5D si2 | 92.6 | 1.9 |  | HEK ZSCAN5D si2 | 147.2 | 9.5 |  | HEK ZSCAN5D si2 | 32.4 | 16.2 |
| HEK Sc Ctrl | 100.0 | 8.7 |  | HEK Sc Ctrl | 100.0 | 7.9 |  | HEK Sc Ctrl | 100.0 | 17.5 |

**Supplementary Table 1C: Densitometric analyses of affected ZSCAN5 family protein expression level after specific ZSCAN5 gene ablation**

After siRNA knockdown, resulting Western Blot was processed and analyzed with ImageJ software to quantitatively evaluate the knock down rates in protein level. Signal intensity values of internal control (TBP) of each sample was used to normalize and calculate the % expression after gene ablation.

|  | Samples density | TBP control  density | Normalization  against TBP control | % Expression |
| --- | --- | --- | --- | --- |
| ZSCAN5A si | 1324.77 | 21704.903 | 0.061035518 | 15.87 |
| Sc ctrl | 9050.255 | 23533.731 | 0.384565244 |  |
| ZSCAN5B si | 3401.79 | 20085.388 | 0.169366407 | 32.97 |
| Sc ctrl | 9608.154 | 18705.702 | 0.513648405 |  |
| ZSCAN5D si | 1718.841 | 25027.711 | 0.068677515 | 19.93 |
| Sc ctrl | 8346.912 | 24229.468 | 0.344494233 |  |

**Supplementary Table 1D: Increased numbers of M-phase cells after ZSCAN5A knockdown**

Stably transfected Dox-inducible HEK-293 cell lines expressing short hairpin RNA targeting ZSCAN5A (ZSCAN5A-Tet-shRNA), or mock transfection control were incubated with 1 µg/mL Dox for 48h on gelatin-coated glass cover slips. Fixed coverslips were stained with mitotic marker anti-Phospho-Histone H3 (Ser10) antibody, and DAPI to visualize mitotic cells using microscopy. The resulting images were processed and total nuclei and mitotic nuclei were counted using Image J software [[53](#_ENREF_53)].

| **Sample** | **Total cells** | **Mitotic cells** | **Ratio of Mitotic Cells** | **Sample** | **Total cells** | **Mitotic cells** | **Ratio of Mitotic Cells** | **p-value** |
| --- | --- | --- | --- | --- | --- | --- | --- | --- |
| 5A_sh with Dox | 6585 | 717 | 0.108883827 | BLANK with Dox | 2652 | 165 | 0.062217195 | 5.04E-12 |
| 5A_sh without Dox | 6533 | 361 | 0.055257921 | BLANK without Dox | 4690 | 409 | 0.087206823 | 4.01E-11 |
