# Supplementary material for: ZSCAN5B and primate-specific paralogs bind RNA polymerase III genes and extra-TFIIIC (ETC) sites to modulate mitotic progression
Source: Oncotarget. 2016 Oct 6;7(45):72571–92. doi: 10.18632/oncotarget.12508 (PMC5340127; doi:10.18632/oncotarget.12508)
Supplement: Supplementary file 7 [file oncotarget-07-72571-s007.docx]

**Supplementary Table 6: DNA sequences of oligonucleotides used**

| Gene expression primers | DNA sequence (5'>>3') | Amplicon size | Description | |  |
| --- | --- | --- | --- | --- | --- |
| ZSCAN5A-F | ACACAAGAGTATTGACGTAACAGGTGA | 498 | human ZSCAN5A transcript detection | |  |
| ZSCAN5A-R | GCTTGGAATTACACGTAAACCTCTTCTCG |  |  | |  |
| ZSCAN5B-F | AAGAGTCCCACAGATCTGGTGAG | 388 | human ZSCAN5B transcript detection | |  |
| ZSCAN5B-R | TGCTTAGCTGGGAAAAATACTTAAATGATTTATTG |  |  | |  |
| ZSCAN5C-F | TTCCAAACAGTCCCACAGGGG | 468 | human ZSCAN5C transcript detection | |  |
| ZSCAN5C-R | CATGCAGCGCATAGGCCTG |  |  | |  |
| ZSCAN5D-F | CTGTGGTCAATTTTCTTGGCAAGGA | 367 | human ZSCAN5D transcript detection | |  |
| ZSCAN5D-R | CCCACCACACCTGTAGGACC |  |  | |  |
| hYWHAZ-F | ACTTTTGGTACATTGTGGCTTCAA | 95 | human internal reference control | |  |
| hYWHAZ-R | CCGCCAGGACAAACCAGTAT |  |  | |  |
| mZscan5b-F | AGCCGCAGAGGATAAATGGG | 211 | mouse Zscan5b transcript detection | |  |
| mZscan5b-R | TGGGCTCATCTCAGGTCTCA |  |  | |  |
| mYwhaz-F | TTGATCCCCAATGCTTCGC | 88 | mouse internal reference control | |  |
| mYwhaz-R | CAGCAACCTCGGCCAAGTAA |  |  | |  |
|  |  |  |  | |  |
| Cell cycle reference gene primers | **DNA sequence (5'>>3')** | **Amplicon size** | **Description** | |  |
| CCNE1-F | GCACTTCAGGGGCGTCGCTG | 234 | G1/S reference marker | |  |
| CCNE1-R | AGCAGGCGCGCAACTGTCTT |  |  | |  |
| RRM2-F | TGGCTCAAGAAACGAGGACTGATGC | 205 | S reference marker | |  |
| RRM2-R | TGAGCTTCACAGGCAAGGCCTC |  |  | |  |
| CDC2-F | ACTGGCTGATTTTGGCCTTGCCA | 243 | G2 reference marker | |  |
| CDC2-R | TTGGGAGTGCCCAAAGCTCTGAAAA |  |  | |  |
| BUB1-F | TGAGGATCTGCCCGCTTCCCA | 210 | G2/M reference marker | |  |
| BUB1-R | GTGGCTGGGGACACCAAAGCTG |  |  | |  |
| PTTG1-F | AGGGACCCCTCAAACAAAAACAGCC | 188 | M/G1 reference marker | |  |
| PTTG1-R | GGGGAGGTGCGCAATCTGGT |  |  | |  |
|  |  |  |  | |  |
| non-tRNA DEG primer | **DNA sequence (5'>>3')** | **Amplicon size** |  | |  |
| RMRP-F | AGGCTACACACTGAGGACTCT | 192 |  | |  |
| RMRP-R | CCTGCGTAACTAGAGGGAGC |  |  | |  |
|  |  |  |  | |  |
| tRNA gene primers  from ref. [[13](#_ENREF_13)] | **DNA sequence (5'>>3')** | **Amplicon size** |  | |  |
| tRNA_R-Chr9-F | CTCTGTGGCGCAAATGGATAG | 54 | tR(TCT):chr9 | |  |
| tRNA_R-Chr9-R | TGACCACACTAGGCTCAG |  |  | |  |
| tRNA_R-Chr17-F | CTCTGTGGCGCAAATGGATAG | 54 | tR(TCT):chr17 | |  |
| tRNA_R-Chr17-R | GAATTGCTCTATYCGTCACTAG |  |  | |  |
| tRNA_I-Chr19-F | TCCAGTGGCGCAATCGGT | 57 | tI(TAT):chr19 | |  |
| tRNA_I-Chr19-R | ATTGCTCCGCTCGCACTGTC |  |  | |  |
| tRNA_Y-Chr2-F | CCTTCGATAGCTCAGTTGGT | 54 | tY(GTA):chr2 | |  |
| tRNA_Y-Chr2-R | TTGCCACGCCCTATCCA |  |  | |  |
|  |  |  |  | |  |
| siRNA (Qiagen) | **siRNA sequence** | **Size** | **siRNA Cat. # (Qiagen)** | |  |
| ZSCAN5A_si4 | CGAGAAGAGGTTTACGTGTAA | 21 | SI00779436 | |  |
| ZSCAN5A_si5 | AAGCTAGTCATCCACAAGAGA | 21 | SI04221826 | |  |
| ZSCAN5B_si1 | ACGTGTGCAATAAATCATTTA | 21 | SI00503300 | |  |
| ZSCAN5D_si2 | CAGGAAGAACCTGAACGAGCA | 21 | SI02804774 | |  |
|  |  |  |  | |  |
| ISH probes | **RNA sequence** | **Size** | **Description** | |  |
| ZSCAN5B-ISH-Probe | CCAGAGTCAGGTCCACATCCTCCACCCCAAGAGACAGTGAATGATGGTTTTTCTCCAATGTTACCAGATGACATATACAC | 80 | ISH probe design to detect human *ZSCAN5B* | |  |
| mZscan5b-ISH-Probe | GAAAACCCACCTGAGAAAGAGAAAGGTGTCTGAGTAATTCTGGATCCAGTTTTTTCCCCTAGAAAGACTGAGATTGAAGTCTTTTCTCAGAGAAATGGAGGTTGGGTTG | 109 | ISH probe design to detect mouse *Zscan5b* | |  |
|  |  |  |  | |  |
| EMSA probes | **DNA sequence (5'>>3')** | | | Size |  |
| STAP2_M2-F | 5’biotin-CGGGTCGGACTCCGCCCCTGCTTCTGA-3’ | | | 27 |  |
| STAP2_M2-R | 5’-TCAGAAGCAGGGGCGGAGTCCGACCCG-3’ | | |  |  |
| STAP2_M1-F | 5’biotin-CTGACCACGCCCCCGCGCCCACCCTCTT-3’ | | | 28 |  |
| STAP2_M1-R | 5’-AAGAGGGTGGGCGCGGGGGCGTGGTCAG-3’ | | |  |  |
| STAP2_M2+M1-F | 5’biotin-CGGGTCGGACTCCGCCCCTGCTTCTGACCACGCCCCCGCGCCCACCCTCTT-3’ | | | 51 |  |
| STAP2_M2+M1-R | 5’-AAGAGGGTGGGCGCGGGGGCGTGGTCAGAAGCAGGGGCGGAGTCCGACCCG-3’ | | |  |  |
|  | **ZSCAN5A shRNA oligonucleotide sequences**  5’ – *Bgl*II – sense – hairpin – antisense – *Xho*I – 3’ | | | **Size** |  |
| ZSCAN5A-Tet-shRNA-sense | GATCCCCGAGAAGAGGTTTACGTGTATTCAAGAGATACACGTAAACCTCTTCTCTTTTTA | | | 60 |  |
| ZSCAN5A-Tet-shRNA-antisense | TCGATAAAAAGAGAAGAGGTTTACGTGTATCTCTTGAATACACGTAAACCTCTTCTCGGG | | | 60 |  |
